# Supplementary material for: Description of the rates, trends and surgical burden associated with revision for prosthetic joint infection following primary and revision knee replacements in England and Wales: an analysis of the National Joint Registry for England, Wales, Northern Ireland and the Isle of Man
Source: BMJ Open. 2017 Jul 10;7(7):e014056. doi: 10.1136/bmjopen-2016-014056 (PMC5541502; doi:10.1136/bmjopen-2016-014056)
Supplement: Supplementary data 1 [file bmjopen-2016-014056supp001.pdf]

Appendix Table 1: Characteristics of patients with a completed or partially-completed two-stage revision total knee replacement †

	Complete*	Only stage two**	Only stage one**
**Primary(n=2,880)**	1,512	792	576
Age(mean, SD)	66(9)	67(9)	67(10)
Female(%)	44	41	43
BMI(mean, SD)	32(6)	32(6)	32(6)
missing BMI (n=)	319	308	204
ASA 1(%)	14	14	10
ASA 2(%)	68	67	64
ASA 3(%)	18	19	25
ASA 4-5(%)	0	0	1
**Revision(n=589)**	309	154	126
Age(mean, SD)	66(10)	66(10)	68(10)
Female(%)	40	41	33
BMI(mean, SD)	32(6)	31(6)	32(6)
missing BMI	63	58	32
ASA 1(%)	14	10	14
ASA 2(%)	67	67	58
ASA 3(%)	19	22	27
ASA 4-5(%)	0	1	1

†2,880 two-stage revision total knee replacement procedures to manage an infection were recorded in the NJR following an index primary knee replacement and 589 following an index aseptic revision surgery.

* Both stage one and stage two procedures of a two-stage revision total knee replacement reported in the NJR.

**Only stage one or stage two procedure of a two-stage revision total knee replacement reported in the NJR.
