# Supplementary figures and images for: Description of the rates, trends and surgical burden associated with revision for prosthetic joint infection following primary and revision knee replacements in England and Wales: an analysis of the National Joint Registry for England, Wales, Northern Ireland and the Isle of Man
Source: BMJ Open. 2017 Jul 10;7(7):e014056. doi: 10.1136/bmjopen-2016-014056 (PMC5541502; doi:10.1136/bmjopen-2016-014056)

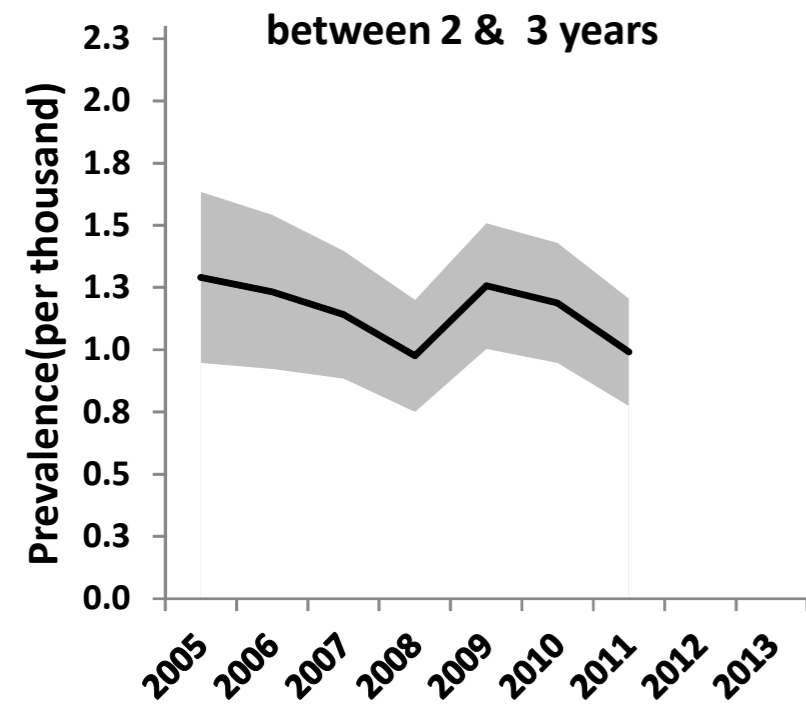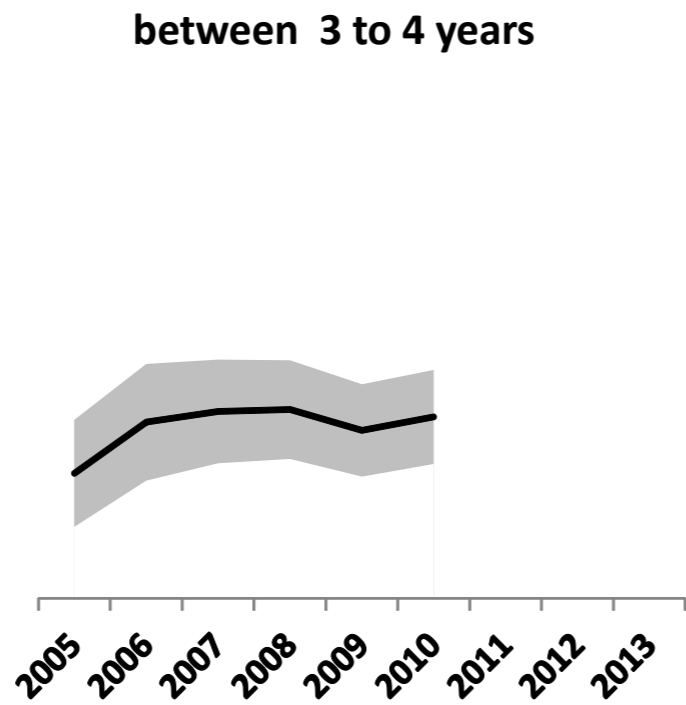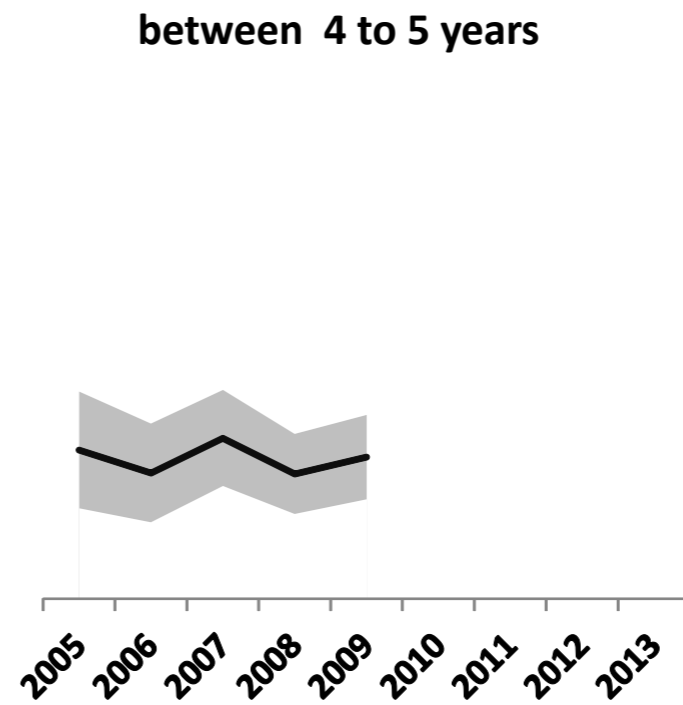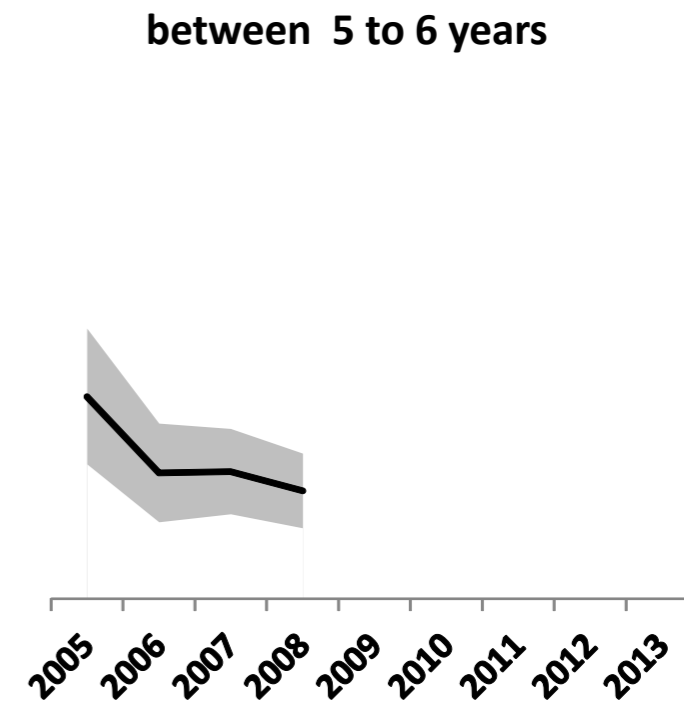

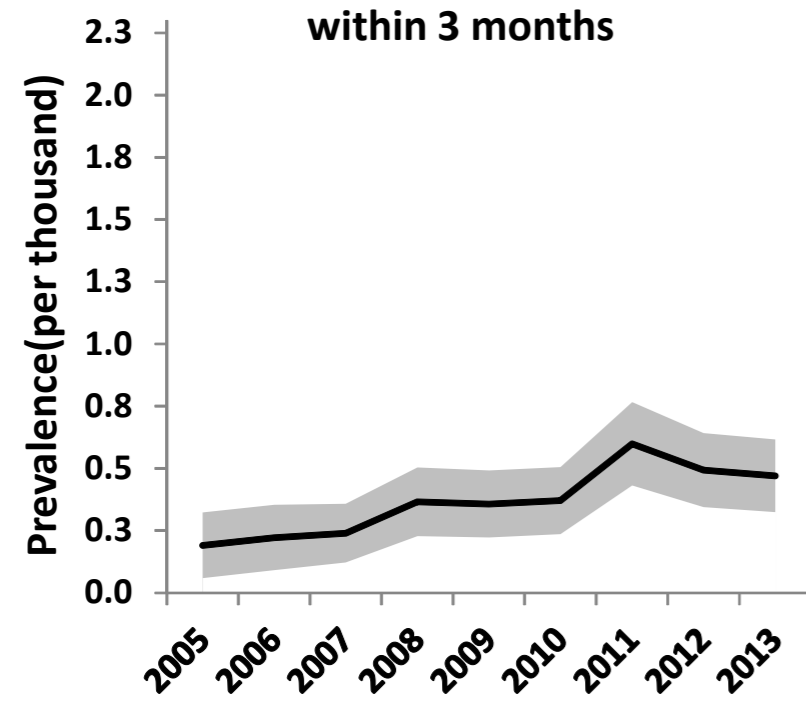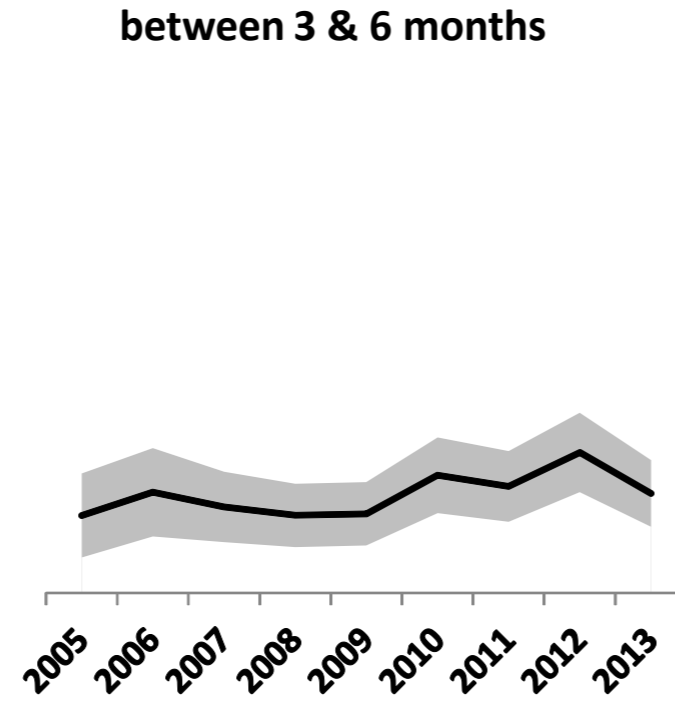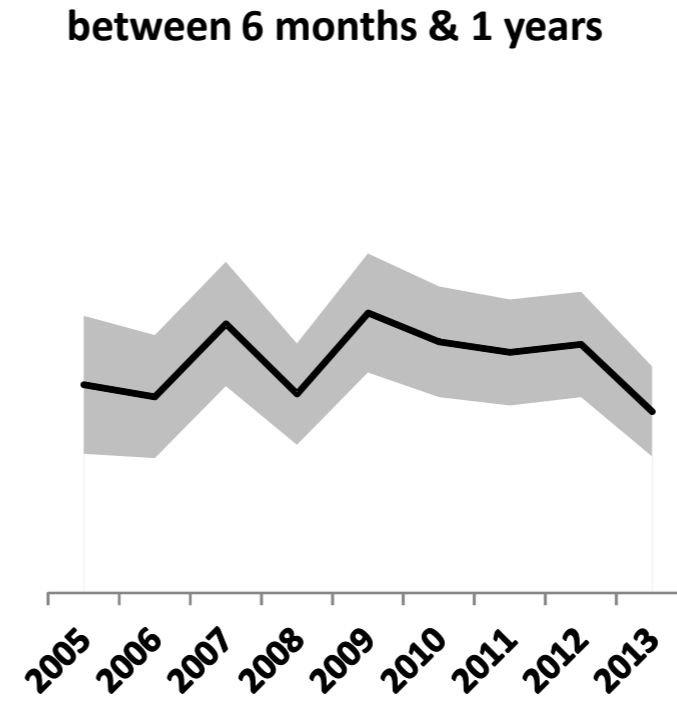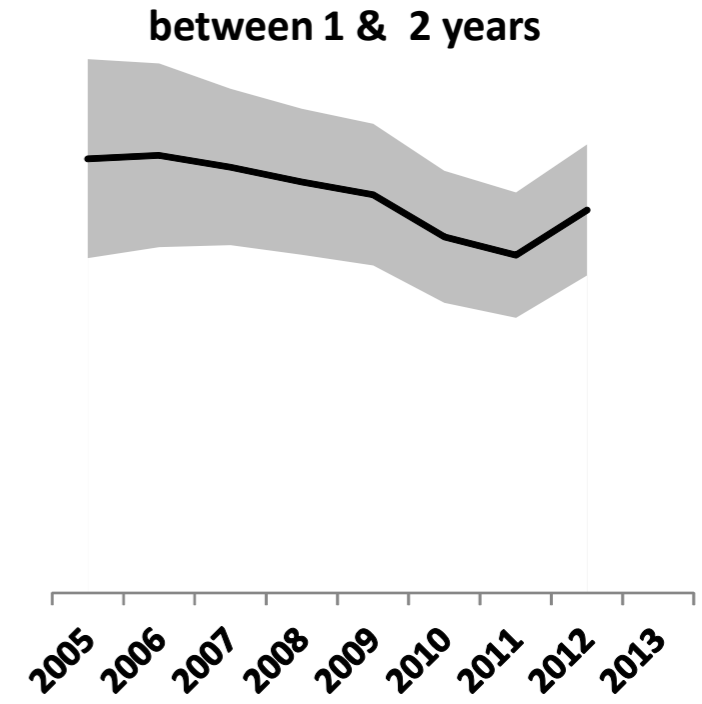

Supplement: Supplementary data 2 [file bmjopen-2016-014056supp002.pdf]
