# Supplementary material for: Description of the rates, trends and surgical burden associated with revision for prosthetic joint infection following primary and revision knee replacements in England and Wales: an analysis of the National Joint Registry for England, Wales, Northern Ireland and the Isle of Man
Source: BMJ Open. 2017 Jul 10;7(7):e014056. doi: 10.1136/bmjopen-2016-014056 (PMC5541502; doi:10.1136/bmjopen-2016-014056)
Supplement: Author's manuscript [file bmjopen-2016-014056.draft_revisions.pdf]

BMJ Open

Revision for prosthetic joint infection following primary and revision knee replacement and its overall surgical burden. An analysis of the National Joint Registry for England, Wales, Northern Ireland and the Isle of Man.

Journal:	*BMJ Open*
Manuscript ID	bmjopen-2016-014056
Article Type:	Research
Date Submitted by the Author:	01-Sep-2016
Complete List of Authors:	Lenguerrand, Erik; University of Bristol School of Clinical Science, School of Clinical Sciences, Musculoskeletal Research Unit<br>Whitehouse, Michael; University of Bristol, Musculoskeletal Research Unit<br>Beswick, Andrew; University of Bristol, School of Clinical Sciences<br>Toms, Andrew ; Royal Devon and Exeter NHS trust<br>Porter, Martyn; Wrightington Hospital<br>Blom, Ashley; University of Bristol, School of Clinical Sciences
**Primary Subject Heading**:	Surgery
Secondary Subject Heading:	Epidemiology
Keywords:	Knee < ORTHOPAEDIC & TRAUMA SURGERY, Primary knee replacement, Revision knee replacement, prosthetic joint infection, Registry

SCHOLARONE™
Manuscripts

For peer review only

Revision for prosthetic joint infection following primary and revision knee replacement and its overall surgical burden. An analysis of the National Joint Registry for England, Wales, Northern Ireland and the Isle of Man.

*Erik Lenguerrand[1], *Michael R Whitehouse[1], Andrew D Beswick[1], Andrew D Toms[2], Martyn L Porter[3], Ashley W Blom[3], on behalf of the National Joint Registry for England, Wales, Northern Ireland and the Isle of Man.

* Authors contributed equally to this work

1. Musculoskeletal Research Unit, School of Clinical Sciences, University of Bristol, Bristol, UK
2. Exeter Knee Reconstruction Unit, Princess Elizabeth Orthopaedic Centre, Royal Devon and Exeter NHS trust, Exeter, UK
3. Centre for Hip Surgery, Wrightington Hospital, Lancashire, WN6 9EP

Correspondence to:
Name: Dr Erik Lenguerrand
Address: Musculoskeletal Research Unit, 1st Floor Learning & Research Building, School of Clinical Sciences, University of Bristol, Southmead Hospital, Bristol, BS10 5NB, UK
Email: erik.lenguerrand@bristol.ac.uk Tel: 0117 414 7865 Fax: 0117 414 7924

Corresponding author:
Name: Prof Ashley W Blom
Address: Musculoskeletal Research Unit, 1st Floor Learning & Research Building, School of Clinical Sciences, University of Bristol, Southmead Hospital, Bristol, BS10 5NB, UK
Email: ashley.blom@nbt.nhs.uk Tel: 0117 414 7865

Co-corresponding author:
Name: Mr Martyn L Porter
Address: Centre for Hip Surgery, Wrightington Hospital, Lancashire, WN6 9EP, UK
Email: martynporter@hipkneeclinic.com Tel: 01257 266 166

Word count: 3893

For peer review only

ABSTRACT (max 300 words)

Objectives

To describe the prevalence rates of revision surgery for the treatment of prosthetic joint infection (PJI) for patients undergoing knee replacement, their time trends, the cumulative incidence function of revision for PJI and estimate the burden of PJI at health service level.

Design

We analysed revision knee replacements performed due to a diagnosis of PJI and the linked index procedures recorded in the National Joint Registry from 2003–2014 for England and Wales. The cohort analysed consisted of 679,010 index primary knee replacements, 33,920 index revision knee replacements and 8,031 revision total knee replacements performed due to a diagnosis of PJI. The prevalence rates, their time-trends investigated by time from index surgery to revision for PJI, cumulative incidence functions, and the burden of PJI (total procedures) were calculated. Overall linear trends were investigated with log-linear regression.

Results

We demonstrated a risk of revision total knee replacement due to PJI of 0·5% following primary and 2.1% following revision knee replacement. The risk of revision due to PJI in the 3 months following primary knee replacement has risen by 2·5-fold (95% CI 1.2–5.3) from 2005–2013 and 7·5-fold (95% CI 1.0–56.1) following revision knee replacement. Over 1,000 procedures/year are performed as a consequence of knee PJI, an increase of 2·8 from 2005-2013. Overall, 74% of revisions were two-stage with an increase in use of single stage from 10.0% in 2005 to 29.0% in 2014.

Conclusions

For peer review only

Although the risk of revision due to PJI following knee replacement is low, it is rising, and coupled with the established and further predicted increased incidence of both primary and revision knee replacement, this represents an increasing and substantial treatment burden for orthopaedic service delivery in England and Wales. This has implications for future service design and the funding of individual and specialist centres.

Keywords: Orthopaedics, Primary knee replacement, Revision knee replacement, prosthetic joint infection, Registry

For peer review only

STRENGHT AND LIMITATIONS OF THIS STUDY

- This study provides updated and nationally representative descriptive evidence for England and Wales on revision for prosthetic joint infection following knee replacements.
- Over 600,000 index primary replacements, 30,000 index revision replacements and 8,000 revision total replacements performed due to a diagnosis of infection are included in the analyses.
- Contemporary evidence regarding changes over time in the risk of revision for prosthetic joint infection, its burden, and the types of revision surgery used for managing prosthetic joint infection is provided.
- The diagnosis of prosthetic joint infection recorded in the registry is not validated against a recognised international standard and may therefore be subject to over or under reporting.
- Not all recorded revisions for prosthetic joint infection had an index primary or aseptic revision total knee replacement recorded in the National Joint Registry.

For peer review only

BACKGROUND

Deep prosthetic joint infection (PJI) is a rare but serious complication of total knee replacement. Patients experience severe pain, functional difficulties, poor quality of life and, if untreated, loss of the affected limb or death. [1-3]

Estimates of the incidence of PJI after primary knee replacement range between 0.85% in Germany, 1.0% in the UK, 1.4% in Finland and 2.2% in the USA.[4-7] In a study from Finland, about 1.14% of patients had a PJI within 2 years of their primary knee replacement with a further 0.27% of patients presenting with PJI later than 2 years after primary surgery.[6] After aseptic revision and more complex knee replacement surgeries, infection rates may be considerably higher.[8] There is a paucity of evidences from UK centres and the available studies are old and based on single-centre data.[4 9]

Bacteria adhere as biofilm to implants and peri-prosthetic tissues making the treatment of PJI difficult.[10] Patients with PJI after knee replacement and their treating surgeons face complex and protracted treatment pathways. Initial treatment may involve surgical debridement, antibiotics and implant retention (DAIR), particularly in early postoperative infections. However about 45-52% of patients receiving DAIR may subsequently need revision of their implants [11] and delays to effective infection control inherent in this process may lead to a poor post replacement outcome.[12]

For the majority of patients with PJI after knee replacement, contemporary management involves surgical revision with extensive debridement and antibiotic treatment with either immediate implant replacement (single-stage) or delayed implant replacement (two-stage).

The two-stage revision procedure was the first method reported for treatment of PJI of the knee that aimed to restore a functioning, painless knee.[13] The period between operations allows targeted antibiotic treatment and monitoring of clinical status and inflammatory markers. However, patients require two distinct planned major surgeries and a period of several months with limited knee function and protracted distress, concern and uncertainty.[14] An alternate one-stage strategy developed by the Endo-Klinik and reported in 1976.[15]. Key to the single-stage revision strategy is extensive debridement, identification of the infecting organisms and appropriate tailored antibiotic treatment.[16]

There has been no randomised trial comparing the outcome of single-stage and two-stage revision for PJI of the knee but evidence from case-series suggests similar rates of re-infection after each treatment.[17] The decision to treat with a single-stage or two-stage procedure may be guided by

For peer review only

microbiological tests and patient health but ultimately the choice of revision method is largely that of the treating surgeon. [2]

The National Joint Registry for England, Wales Northern Ireland and the Isle of Man (NJR) is the largest joint replacement database in the world and has collected data since April 2003.[18]

We analysed this dataset to assess the burden of revision surgery for knee PJI between April 2003 and December 2013. In this period, data was available from England and Wales. Our specific aims were to:

- Present the surgeon's perspective by describing the prevalence rates of revision surgery for the treatment of knee PJI following primary and revision surgery for aseptic indications, and their time trends broken down by time from index surgery to revision for infection.
- Present the patient's perspective by deriving cumulative incidence functions by type of index surgery.
- Estimate the burden of revision surgery for knee PJI at a health service level by accounting for all of the registered revisions and re-revision surgeries performed for knee PJI.

For peer review only

METHODS

Data source

In this observational study, we report analyses of data from the NJR.[18] The registry was established in 2003 and includes details of primary and revision knee replacements performed in England and Wales. Data entry for Northern Ireland and the Isle of Man did not commence until 2013 and 2015 respectively and data linkage for those periods is limited, therefore they are excluded from this analysis.

We grouped procedures as index knee replacements and subsequent revision surgeries for treatment of knee PJI. All index surgeries performed between 1st April 2003–31st December 2013 were included (to allow a minimum 12-months follow-up). All revision surgeries performed as a consequence of PJI between 1st January 2003 and 31st December 2014 were considered.

Index surgeries

Index surgeries included all primary procedures and all revision procedures performed for an indication other than infection. The index revision procedures have been labelled "aseptic revision" procedures to indicate they were not performed due to PJI. Revisions not performed for PJI but that were performed on a knee previously operated on due to PJI were not considered as index surgeries. PJI is selected as the indication for the revision procedure at the time of surgery by the responsible surgeon. Revision procedures were classified as a single stage, a stage one of a two-stage revision, a stage two of a two-stage revision procedure, a conversion to arthrodesis, or an amputation.

Missing procedures

There were 2,833 two-stage revision procedures recorded where the index surgery was a primary knee replacement, in 792 (28%) of these, only a stage two of a two-stage procedure was recorded in the NJR. This was also observed for 154 (27%) of the 576 two-stage revision procedures following an index aseptic revision procedure. Patients with incompletely registered two-stage procedures did not differ from those with complete information in terms of gender, BMI, and ASA grade (see appendix Table 1). For index primary knee replacement, patients with incomplete information were on average one year

For peer review only - http://bmjopen.bmj.com/site/about/guidelines.xhtml

For peer review only

older than those with complete information (p=0.05). For aseptic revision surgeries, no age difference was observed between the two groups.

For incomplete procedures, the date of the stage one of a two-stage revision procedure was estimated. Firstly we derived the relative weight of time elapsed between the index and stage one of a two-stage procedure by year and type of index surgery using patients with complete information: 100x(length of time$_{\text{index surgery- 1st stage}}$/length of time $_{\text{index surgery- 2nd stage}}$). We applied these weights to the length of time observed between the index and stage two of a two-stage procedure for those with incomplete information to obtain an estimate for the time between the index surgery and stage one of the revision surgery.

Statistical analyses

Analyses were performed with Stata SE 13·1 (StataCorp LP, TX, USA).

Surgeon perspective

Prevalence rates of index surgeries performed between 2003–2013 and subsequently revised for infection between 2003–2014 were derived by year and type of index surgery. The prevalence rates were plotted by time from index surgery to revision for infection (≤3 months, 3–6 months, 6 months–1 year, 1–2 years, 2–3 years, 3–4 years, and 5–6 years). For two-stage surgeries, the date of stage one of a two-stage revision was selected to indicate the date of revision for PJI. Log-linear regression, using the year of the index knee replacement as a continuous independent factor, was used to investigate overall linear trends between 2005–2013. This period was selected as over 85% of knee replacements performed in 2005 and over 99% of those performed from 2007 onwards had been recorded in the NJR; prior to 2005, the data capture of the NJR was <75%.[19] When evidence of a time-trend was identified (year of surgery, p≤0·05), the year of surgery was re-considered as a categorical variable using 2005 as the reference period. Estimated relative risk and related 95% confidence intervals (95%CI) quantified the relative increase in rates between the period of interest and 2005.

For peer review only - http://bmjopen.bmj.com/site/about/guidelines.xhtml

Patient perspective

Cumulative incidence functions [20-22] were derived by type of index surgery (primary or aseptic revision). These provide the probability of being revised as a consequence of PJI within a specific time period following the index surgery while accounting for the time patients were at risk of being revised for a PJI and the competing risks of death and revision for an aseptic indication.

Healthcare service perspective

The overall burden of revision surgery for knee PJI was analysed using all revision procedures performed between 2003–2014 for an indication of PJI as well as any subsequent re-revision procedures for PJI or another indication.

Typically, knee replacement survivorship is 10 years or more following surgery,[18] re-revision procedures performed for another indication than PJI during the observation period are therefore considered as a consequence of the earlier management of PJI.

Revision procedures for an indication of PJI with no index procedure recorded in the NJR (n=3,064, Figure 1) and their re-revision procedures were also accounted. Contrary to the previous analyses in which index surgeries performed in 2014 and their revisions procedures were not considered to allow for a minimum of one-year post-operative follow-up, revisions for PJI relating to index knee replacements performed in 2014 (n=122) were included in this analysis.

The overall burden was reported by the year and type of revision surgery. Stage one of a two-stage revision and stage two of a two-stage revision were considered as one procedure to avoid double counting. The same strategy was also used to account for multiple stage ones of a two-stage revision procedure.

For peer review only

RESULTS

Surgeon perspective

There were 679,010 primary and 33,920 aseptic revision knee replacements recorded in the NJR for England and Wales between April 2003 and December 2013 (Table 1).

As shown in Figure 1, a total of 3,659 patients required at least one revision surgery due to PJI (4,026 procedures) following a primary knee replacement. A further 717 patients required a subsequent revision for PJI (789 procedures) following an aseptic revision knee replacement. For 2,918 patients who underwent revision for PJI (3,216 procedures), no index surgery was recorded.

<Figure 1 here>

Figure 1. Description of procedures recorded in the National Joint Registry.

Of the primary knee replacements recorded in the NJR, 0·54% (95%CI 0.52-0.56) were subsequently revised due to PJI (Table 1). Of the aseptic revision knee replacements, 2·11% (95%CI 1.96-2.23) were subsequently revised due to PJI.

For peer review only

Table 1. Primary and aseptic revision knee replacement procedures revised for a prosthetic knee joint infection (PJI).

Year of index procedure	Index primary replacement			Index aseptic revision replacement		
	Total N=	Revised for PJI n=	Prevalence rate Per 1,000, 95%CI	Total N=	Revised for PJI n=	Prevalence rate Per 1,000, 95%CI
**Total**	**679,010**	**3,659**	**5.39(5.21, 5.56)**	**33,920**	**717**	**21.14(19.61, 22.67)**
2003	13,547	112	8.27(6.74, 9.79)	534	24	44.94(27.37, 62.52)
2004	27,706	184	6.64(5.68, 7.60)	1,001	24	23.98(14.50, 33.45)
2005	41,848	314	7.50(6.68, 8.33)	1,612	41	25.43(17.75, 33.12)
2006	49,482	346	6.99(6.26, 7.73)	2,094	55	26.27(19.42, 33.12)
2007	66,618	471	7.07(6.43, 7.71)	2,847	60	21.07(15.80, 26.35)
2008	73,859	443	6.00(5.44, 6.55)	3,538	87	24.59(19.49, 29.69)
2009	75,612	465	6.15(5.59, 6.71)	3,884	91	23.43(18.67, 28.19)
2010	78,276	423	5.40(4.89, 5.92)	4,196	102	24.31(19.65, 28.97)
2011	81,795	378	4.62(4.16, 5.09)	4,336	81	18.68(14.65, 22.71)
2012	85,161	341	4.00(3.58, 4.43)	5,115	93	18.18(14.52, 21.84)
2013	85,106	182	2.14(1.83, 2.45)	4,763	59	12.39(9.25, 15.53)

For peer review only

For peer review only - http://bmjopen.bmj.com/site/about/guidelines.xhtml

Figure 2 shows trends in revision for PJI in the 2 years after primary knee replacement. Revision rates within 3 months of the index procedure increased over time, with the rate in 2013 over twice that in 2005, RR 2·46 (95%CI 1.15-5.25; time trend p<0·0001). No time-trends for revision rates between 2005 and 2013 were apparent in the periods 3–6 months, 6 months–1 year and 1–2 years. Other than the rate of revision for PJI between 5–6 years which decreased over time, RR 0·53 (95%CI 0·33-0·86; time trend p=0.018), there was no evidence of time-trends for the rates of late revision for PJI (Web appendix Figure1).

<Figure 2 here>

Figure 2. Prevalence (95%CI) of revision for prosthetic joint infection within 2 years of the index primary knee replacement.

Trends in subsequent revision of aseptic revision knee replacements for PJI are shown in Figure 3 and Web appendix Figure 2. The proportion of aseptic revisions revised for PJI within 3 months of the procedure increased over time, RR 7·47 (95%CI 1·00-56·12; time trend p=0·001 for 2013 compared to 2006). No important differences were noted in the rates of revision for PJI performed at any time beyond 3 months from the index revision surgery (all time trend p-values >0·1) with the exception of revision for infection between 2–3 years for which the rates decreased over time, RR 0.40 (95%CI 0.18-0.88; time trend p=0.008 for 2011 compared to 2005).

<Figure 3 here>

Figure 3. Prevalence (95%CI) of revision for prosthetic joint infection within 2 years of the index aseptic revision knee replacement.

Patient perspective

Figure 4 shows the probability over time of revision for PJI in patients with an index primary or aseptic revision while accounting for the risk of death and revision for any indication other than PJI. The probability of revision for PJI at one year following a primary knee replacement was 0·17% (95%CI 0·16-0·18) compared to 0·76% (95%CI 0·68-0·86) following an aseptic revision knee replacement. At two years the probability was 0·32% (95%CI 0·31-0·34) and 1·44% (95%CI 1·31-1·57) respectively, and at five years it was 0·56% (95%CI 0·54-0·58) and 2·41% (95%CI 2·23-2·61) respectively. The probability

For peer review only

of revision for infection within the first ten years following primary knee replacement was 0·75% (95%CI 0·72-0·78) and 3·13% (95%CI 2·81-3·49) following an aseptic revision.

<Figure 4 here>

Figure 4. Cumulative incidence function of revision for prosthetic joint infection following index primary and aseptic revision knee replacement.

Healthcare service perspective

Table 2 summarises the revision surgeries for the management of PJI and subsequent re-revisions (including repeated procedures to manage PJI and other revision procedures) performed after primary and aseptic revision knee replacement by the year the (re-)revision was performed.

The absolute number of procedures performed as a consequence of PJI in England and Wales has increased from 378 in 2005 to 1,055 in 2013, a relative increase of 2·8 fold. This is higher than the 2.1 fold increase in primary knee replacements over the same period, but similar to the 2·9 increase in aseptic revision surgeries between 2005–2013 (table 1).

Overall, 74% of revisions were conducted with a two-stage procedure but the use of single-stage revision for PJI has increased from 10.0% in 2005 to 29.3% in 2014.

For peer review only

Table 2. Revision procedures performed as a consequence of prosthetic joint infection (PJI) by type and year of procedure *

Year of revision procedure	Total N=	Single stage n=, (%)	Two-stage n=, (%)	Other† n=, (%)
**Total**	**8,658**	**2,139(24.7)**	**6,383(73.7)**	**136(1.6)**
2003	127	126(99.2)	1(0.8)	0(0.00)
2004	239	66(27.6)	170(71.1)	3(1.3)
2005	378	38(10.0)	336(88.9)	4(1.1)
2006	486	66(13.6)	414(85.2)	6(1.2)
2007	666	92(13.8)	560(84.1)	14(2.1)
2008	786	159(20.2)	612(77.9)	15(1.9)
2009	857	187(21.8)	657(76.7)	13(1.5)
2010	941	237(25.1)	695(73.9)	9(1.0)
2011	1,014	254(25.0)	742(73.2)	18(1.8)
2012	1,052	289(27.5)	740(70.3)	23(2.2)
2013	1,057	316(29.9)	722(68.3)	19(1.8)
2014	1,055	309(29.3)	734(69.6)	12(1.1)

*This table reports the revision procedures performed after any index surgery revised as a consequence of PJI including subsequent re-revision procedures whether performed to manage an infection or not. It also includes the revision procedures performed between 2003 and 2014 on 3,064 patients with prosthetic joint infection but with no index procedure documented in the NJR.
† Conversion to arthrodesis or amputation

DISCUSSION

This study is the largest to date investigating the treatment of PJI after knee replacement. It includes 712,930 primary and aseptic revision procedures and 7,294 patients with revision for the treatment of PJI. We have shown that the probability of undergoing a revision for PJI is higher following aseptic revision knee replacement than for primary knee replacement and that the relative risk of undergoing revision for PJI within 3 months of surgery has risen over time for primary and aseptic revision procedures. The total burden of treating PJI of the knee has risen substantially, with over 1,000 procedures performed per year from 2011 onwards, which mirrors the greater rise in aseptic revision surgery compared to primary knee replacements.[18] We have identified an increased use of single stage as compared to two-stage revision for PJI of the knee. The current evidence base shows that there is no difference in reinfection rates following revision for PJI of the knee by single stage or two-stage surgery, suggesting that the utilisation of single stage revision surgery could be safely increased. [17]

For peer review only

Strengths and weaknesses

The NJR is an established, large, prospective arthroplasty register with comprehensive coverage of procedures undertaken, which is reassuring in terms of the generalisability of the data generated by the study. The data is observational and prospectively entered into the registry which contains limitations. The recording of PJI as an indication for surgery is according to the opinion of the treating surgeon at the time of surgery and, as such, the diagnosis has not necessarily been referenced against a gold standard set of criteria and hence may be subject to misdiagnosis. Whilst the NJR now represents a mature dataset with long term follow up, there are still a substantial number of procedures undergoing revision knee replacement where there is no record of a linked index primary or revision knee replacement or where there are an incomplete set of procedures recorded for two stage revision surgery. This may be a reflection of the high survivorship of knee replacement for 10 years or more following surgery,[18] the fact that some PJI is acquired late and that not every case performed since 2003 in England and Wales has been recorded in the NJR. Given there was no difference between patients with complete two-stage episodes recorded and those with partial episodes recorded in terms of age, gender, BMI and ASA grade, we have assumed that the time interval of the former is generalisable to the latter, which may not be the case. The recording of debridements with retention of non-modular components (DAIR procedures) [2] may not be fully captured by registries. Currently in the NJR, the only option to record such procedures where only modular components are performed is to record them as single stage revision surgeries which are not distinct from single stage surgeries where the femoral and tibial components are revised, [23] a potential weakness of the data collection forms as this may be subject to different interpretations by surgeons despite the fact that recording of procedures in which any component is removed or inserted is mandatory.

Comparison with other studies

Revision for infection is a rare complication of knee replacement. Our findings in this respect are consistent with previous studies from England and Wales [4 9] although the reported prevalence rates were higher in previous studies (around 1% at 5 years following primary replacement compared to

For peer review only

0.5% in our study). Our study was not contemporaneous with those (index surgeries performed between 1987-2001 [9] or 1993-1996 [4]) and the considered sample size in them was smaller (n=4,788 and n=931). Evidence from other countries shows similar prevalence rates of revision for infection (rates ranging from 0.4% to 2.2%).[5-7 24 25] Studies which report incidence rates typically include any type of operations for the management of infection (not just those where implants are removed or changed) and this may be why they show higher rates (0.9% to 1.4% between 1 and 5 years postoperatively).[6 26]

We have demonstrated a substantial time trend of an increased risk of revision for PJI within the first 3 months of an index primary or aseptic revision knee replacement being performed. This phenomenon is likely to be multifactorial. Factors that could lead to an increased diagnosis of PJI in this period and hence increased risk of revision include the increased accuracy of tests available to clinicians for the diagnosis of PJI,[27] the increased risk factors for PJI amongst the population undergoing knee replacement population (such as elevated body mass index [28]) and increased bed occupancy within the healthcare setting in which these procedures were performed.[29] There have been similar findings in other countries that could not be accounted for by risk factors recorded in those registries, suggesting this trend could reflect an actual general increase in the risk of PJI. [30]

We have shown a greater risk of revision for PJI following aseptic revision knee replacement (3.1% at 10 years) compared to primary knee replacement (0·8% at 10 years) consistent with the findings of previous cohort studies.[4 25 26 31-33] Previous surgery is a known risk factor for PJI, [34] this may because of further bacterial contamination or increased length of surgery. Revision knee replacement tends to be performed in a population with increased host [35] and procedure risk factors for infection or further revision [36] and involve the introduction of a greater volume of prosthetic material and additional adjuncts such as bone graft that may present a favourable environment for bacterial colonisation and subsequent PJI. [37]

Implications for clinicians and policymakers

The current rate of surgical revision of PJI of the knee stands at approximately 1000 cases per year across the NJR. This represents a significant healthcare burden which has more than doubled over the

For peer review only

last decade, exceeding the rate of increase in primary knee replacements. The cost of revision knee replacement due to PJI is more than three times that of primary knee replacement and over twice that of aseptic revision. [38] In the National Health Service, the costs associated with revision knee replacement due to PJI have been shown to be in excess of £30,000 per case, greater than other indications for revision,[39] even before accounting for costs associated with litigation. Single stage revision offers an advantage over the two-stage approach in terms of both patient and surgeon derived utility values in both the short and long term in the hip [40] and may be associated with superior functional outcomes in the knee [41] suggesting this increased burden could be ameliorated by the increased use of a single stage revision strategy.

Unanswered questions and future research

The observed trend of an increase in the risk of infection in the first 3 months following primary and aseptic revision total knee replacement is not currently well understood. Identification of the risk factors associated with this phenomenon and modifiable risk factors for PJI may help to reduce this risk. Although the utilisation of single stage revision knee replacement for the treatment of PJI has increased, given the current evidence base shows equivalent reinfection rates following single stage compared to two-stage revision surgery for PJI, it would appear the utilisation of this technique could be further increased to alleviate the burden of PJI management. Given the increasing burden of treating PJI, this may help ameliorate the increase in resources that will otherwise be required to treat this condition [42]. There is currently no data available from randomised controlled trials looking at the outcome of these two treatment strategies and such evidence is required to support the decision making process in the planning and treatment of these cases.

For peer review only

DECLARATIONS

Acknowledgments

We thank the patients and staff of all the hospitals who have contributed data to the National Joint Registry. We are grateful to the Healthcare Quality Improvement Partnership, the National Joint Registry Steering Committee, and staff at the National Joint Registry for facilitating this work.

Competing interest

MP is the Medical Director of the National Joint Registry and also acts as Chair of the Programme Steering Committee for the National Institute for Health Research (NIHR) INFORM program (PGfAR program: RP-PG-1210-12005). The other authors have no conflicts of interest.

Funding statement

This article presents independent research funded by the National Institute for Health Research (NIHR) under its Programme Grants for Applied Research program (RP-PG-1210-12005). The funders had no role in study design, data collection and analysis, decision to publish, or preparation of the manuscript.

Authors' contributions

EL, MRW, ADB and AWB designed the study. The data were extracted by Northgate (Hemel Hempstead, UK). EL, ADB, MRW, and AWB reviewed the published work. EL conducted the statistical analyses. All authors interpreted data and wrote the report. EL had full access to all the data and AWB is the guarantor.

Disclaimer

The views expressed represent those of the authors and do not necessarily reflect those of the National Joint Registry Steering Committee or Healthcare Quality Improvement Partnership, who do not vouch for how the information is presented.

For peer review only

The views expressed in this article are those of the authors and not necessarily those of the NHS, the NIHR, or the Department of Health.

Ethic approval and consent to participate

Patient consent was obtained for data collection by the National Joint Registry. According to the specifications of the NHS Health Research Authority, separate informed consent and ethical approval were not required for the present study.

Availability of data and materials

Access to the data analysed in this study required permission from the National Joint Registry for England, Wales and Northern Ireland Research Sub-committee. http://www.njrcentre.org.uk/njrcentre/Research/Researchrequests/tabid/305/Default.aspx contains information on research data access request to the National Joint Registry.

For peer review only

References

1. Cahill JL, Shadbolt B, Scarvell JM, et al. Quality of life after infection in total joint replacement. J Orthop Surg 2008;**16**(1):58-65.

2. Gehrke T, Alijanipour P, Parvizi J. The management of an infected total knee arthroplasty. Bone Joint J 2015;**97-B**(10 Suppl A):20-9, doi: 10.1302/0301-620X.97B10.36475.

3. Zmistowski B, Karam JA, Durinka JB, et al. Periprosthetic joint infection increases the risk of one-year mortality. J. Bone Joint Surg. Am. 2013;**95**(24):2177-84, doi: http://dx.doi.org/10.2106/JBJS.L.00789.

4. Blom AW, Brown J, Taylor AH, et al. Infection after total knee arthroplasty. J. Bone Joint Surg. Br. 2004;**86-B**(5):688-91.

5. Meyer E, Weitzel-Kage D, Sohr D, et al. Impact of department volume on surgical site infections following arthroscopy, knee replacement or hip replacement. BMJ Qual Saf 2011;**20**(12):1069-74, doi: 10.1136/bmjqs.2011.051250.

6. Huotari K, Peltola M, Jamsen E. The incidence of late prosthetic joint infections: a registry-based study of 112,708 primary hip and knee replacements. Acta Orthop. 2015;**86**(3):321-5, doi: 10.3109/17453674.2015.1035173.

7. Kurtz SM, Lau E, Watson H, et al. Economic burden of periprosthetic joint infection in the United States. J. Arthroplasty 2012;**27**(8 Suppl):61-5, doi: 10.1016/j.arth.2012.02.022.

8. Mortazavi SM, Schwartzenberger J, Austin MS, et al. Revision total knee arthroplasty infection: incidence and predictors. Clin. Orthop. Relat. Res. 2010;**468**(8):2052-9, doi: 10.1007/s11999-010-1308-6.

9. Phillips JE, Crane TP, Noy M, et al. The incidence of deep prosthetic infections in a specialist orthopaedic hospital: a 15-year prospective survey. J. Bone Joint Surg. Br. 2006;**88**(7):943-8.

10. Arnold WV, Shirtliff ME, Stoodley P. Bacterial biofilms and periprosthetic infections. J. Bone Joint Surg. Am. 2013;**95**(24):2223-9.

11. Romano CL, Manzi G, Logoluso N, et al. Value of debridement and irrigation for the treatment of peri-prosthetic infections. A systematic review. Hip Int. 2012;**22 Suppl 8**:S19-24, doi: http://dx.doi.org/10.5301/HIP.2012.9566.

12. Sherrell JC, Fehring TK, Odum S, et al. Fate of two-stage reimplantation after failed irrigation and debridement for periprosthetic knee infection. Clin. Orthop. Relat. Res. 2011;**469**(1):18-25.

For peer review only

13. Insall JN, Thompson FM, Brause BD. Two-stage reimplantation for the salvage of infected total knee arthroplasty. J. Bone Joint Surg. Am. 1983;**65**(8):1087-98.

14. Moore AJ, Blom AW, Whitehouse MR, et al. Deep prosthetic joint infection: a qualitative study of the impact on patients and their experiences of revision surgery. BMJ Open 2015;**5**(12):e009495, doi: 10.1136/bmjopen-2015-009495.

15. von Foerster G, Kluber D, Kabler U. Mid- to long-term results after treatment of 118 cases of periprosthetic infections after knee joint replacement using one-stage exchange surgery. Orthopade 1991;**20**(3):244-52.

16. Tibrewal S, Malagelada F, Jeyaseelan L, et al. Single-stage revision for the infected total knee replacement: results from a single centre. Bone Joint J 2014;**96-B**(6):759-64, doi: 10.1302/0301-620X.96B6.33086.

17. Kunutsor SK, Whitehouse MR, Lenguerrand E, et al. Re-Infection Outcomes Following One- And Two-Stage Surgical Revision of Infected Knee Prosthesis: A Systematic Review and Meta-Analysis. PLoS One 2016;**11**(3):e0151537, doi: 10.1371/journal.pone.0151537.

18. *National Joint Registry for England, Wales and Northern Ireland: 12th Annual Report*. Hemel Hempstead: NJR Centre 2015.

19. *National Joint Registry for England and Wales: 9th Annual Report.* Hemel Hempstead: NJR Centre, 2012.

20. Coviello V, M. B. Cumulative incidence estimation in the presence of competing risks. Stata Journal 2004(4):103–12.

21. Gillam MH, Ryan P, Graves SE, et al. Competing risks survival analysis applied to data from the Australian Orthopaedic Association National Joint Replacement Registry. Acta Orthop. 2010;**81**(5):548-55, doi: 10.3109/17453674.2010.524594.

22. Ranstam J, Robertsson O. Statistical analysis of arthroplasty register data. Acta Orthop. 2010;**81**(1):10-4, doi: 10.3109/17453671003587168.

23. National Joint Registry. Knee revision procedure form MDS version 6. Secondary Knee revision procedure form MDS version 6 2016. http://www.njrcentre.org.uk/njrcentre/Portals/0/Documents/England/Data%20collection%20forms/MDSv6.0_K2_V004.pdf.

24. Jamsen E, Varonen M, Huhtala H, et al. Incidence of prosthetic joint infections after primary knee arthroplasty. J. Arthroplasty 2010;**25**(1):87-92, doi: 10.1016/j.arth.2008.10.013.

For peer review only

25. Peersman G, Laskin R, Davis J, et al. Infection in total knee replacement: a retrospective review of 6489 total knee replacements. Clin. Orthop. Relat. Res. 2001(392):15-23.

26. Jamsen E, Huotari K, Huhtala H, et al. Low rate of infected knee replacements in a nationwide series--is it an underestimate? Acta Orthop. 2009;**80**(2):205-12, doi: 10.3109/17453670902947432.

27. Deirmengian C, Kardos K, Kilmartin P, et al. Combined measurement of synovial fluid alpha-Defensin and C-reactive protein levels: highly accurate for diagnosing periprosthetic joint infection. J. Bone Joint Surg. 2014;**96**(17):1439-45, doi: http://dx.doi.org/10.2106/JBJS.M.01316.

28. Paxton EW, Inacio MC, Khatod M, et al. Risk calculators predict failures of knee and hip arthroplasties: findings from a large health maintenance organization. Clin. Orthop. Relat. Res. 2015;**473**(12):3965-73, doi: 10.1007/s11999-015-4506-4.

29. Kaier K, Mutters NT, Frank U. Bed occupancy rates and hospital-acquired infections--should beds be kept empty? Clin. Microbiol. Infect. 2012;**18**(10):941-5, doi: 10.1111/j.1469-0691.2012.03956.x.

30. Kurtz SM, Lau E, Schmier J, et al. Infection burden for hip and knee arthroplasty in the United States. J. Arthroplasty 2008;**23**(7):984-91, doi: 10.1016/j.arth.2007.10.017.

31. Bohl DD, Samuel AM, Basques BA, et al. How much do adverse event rates differ between primary and revision total joint arthroplasty? J. Arthroplasty 2015, doi: 10.1016/j.arth.2015.09.033.

32. Pugely AJ, Martin CT, Gao Y, et al. The incidence of and risk factors for 30-day surgical site infections following primary and revision total joint arthroplasty. J. Arthroplasty 2015;**30**(9 Suppl):47-50, doi: 10.1016/j.arth.2015.01.063.

33. Rasouli MR, Restrepo C, Maltenfort MG, et al. Risk factors for surgical site infection following total joint arthroplasty. J. Bone Joint Surg. Am. 2014;**96**(18):e158, doi: 10.2106/jbjs.m.01363.

34. Tayton ER, Frampton C, Hooper GJ, et al. The impact of patient and surgical factors on the rate of infection after primary total knee arthroplasty : an analysis of 64 566 joints from the New Zealand Joint Registry. Bone Joint J 2016;**98-B**(3):334-40, doi: 10.1302/0301-620X.98B3.36775.

35. Nichols CI, Vose JG. Clinical Outcomes and Costs Within 90 Days of Primary or Revision Total Joint Arthroplasty. J. Arthroplasty 2016, doi: 10.1016/j.arth.2016.01.022.

36. Mahomed NN, Barrett J, Katz JN, et al. Epidemiology of total knee replacement in the United States Medicare population. J. Bone Joint Surg. Am. 2005;**87**(6):1222-8.

For peer review only - http://bmjopen.bmj.com/site/about/guidelines.xhtml

37. Hartley RC, Barton-Hanson NG, Finley R, et al. Early patient outcomes after primary and revision total knee arthroplasty. A prospective study. J. Bone Joint Surg. Br. 2002;**84**(7):994-9.

38. Lavernia C, Lee DJ, Hernandez VH. The increasing financial burden of knee revision surgery in the United States. Clin. Orthop. Relat. Res. 2006;**446**:221-6.

39. Kallala RF, Vanhegan IS, Ibrahim MS, et al. Financial analysis of revision knee surgery based on NHS tariffs and hospital costs: does it pay to provide a revision service? Bone Joint J 2015;**97-B**(2):197-201, doi: 10.1302/0301-620x.97b2.33707.

40. Wolf CF, Gu NY, Doctor JN, et al. Comparison of one and two-stage revision of total hip arthroplasty complicated by infection: a Markov expected-utility decision analysis. J. Bone Joint Surg. Am. 2011;**93-A**(7):631-9.

41. Nagra NS, Hamilton TW, Ganatra S, et al. One-stage versus two-stage exchange arthroplasty for infected total knee arthroplasty: a systematic review. Knee Surg. Sports Traumatol. Arthrosc. 2015.

42. Patel A, Pavlou G, Mujica-Mota RE, et al. The epidemiology of revision total knee and hip arthroplasty in England and Wales: a comparative analysis with projections for the United States. A study using the National Joint Registry dataset. Bone Joint J 2015;**97-B**(8):1076-81, doi: 10.1302/0301-620X.97B8.35170.

For peer review only

For peer review only - http://bmjopen.bmj.com/site/about/guidelines.xhtml

LIST OF ABBREVIATIONS

PJI: Prosthetic joint infection;

NJR: National Joint Registry for England, Wales and Northern Ireland

BMI: Body Mass Index

ASA grade: American Society of Anesthesiologists grade

RR: Rate Ratio;

95% CI: 95% Confidence Interval

ADDITIONAL FILES

Web appendix Table 1: Characteristics of patients with a completed or partially-completed two-stage revision total knee replacement

FIGURE LEGENDS

Figure 1: Description of procedures recorded in the National Joint Registry.

Figure 2: Prevalence (95% CI) of revision for prosthetic joint infection within 2 years from the index primary surgery.

Figure 3. Prevalence (95% CI) of revision for prosthetic joint infection within 2 years from the index aseptic revision surgery.

Figure 4 : Cumulative Incidence Function of revision for prosthetic joint infection following index primary and aseptic revision knee replacement.

ADDITIONAL FIGURE LEGENDS

Web Appendix Fig 1. Prevalence (95% CI) of revision for prosthetic joint infection within 2 to 6 years

For peer review only

from the index primary surgery.

Web appendix Fig 2. Prevalence (95% CI) of revision for prosthetic joint infection within 2 to 6 years from the index aseptic revision surgery.

For peer review only

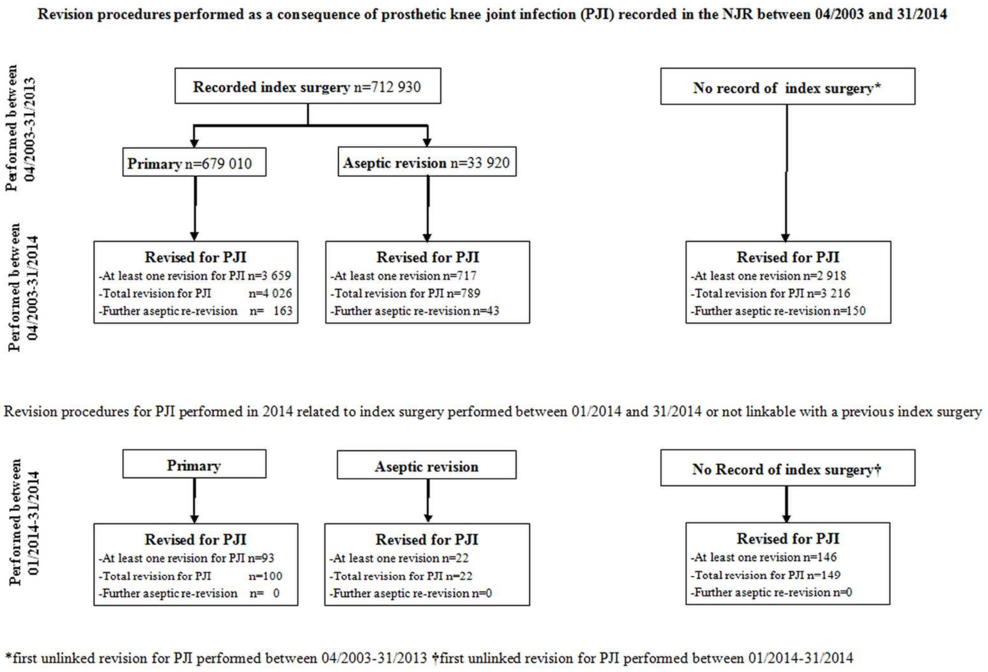

Figure 1
Figure 1
160x107mm (300 x 300 DPI)

For peer review only - http://bmjopen.bmj.com/site/about/guidelines.xhtml

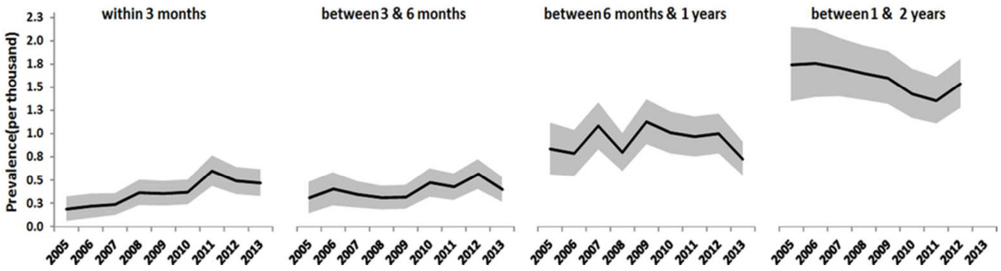

Figure 2
Figure 2
40x10mm (600 x 600 DPI)

For peer review only

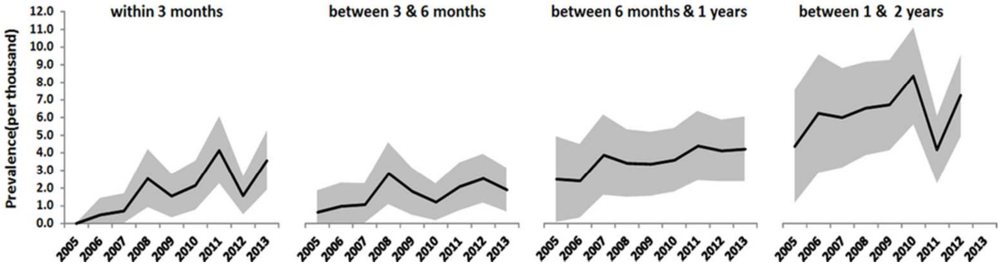

Figure 3
Figure 3
40x10mm (600 x 600 DPI)

For peer review only

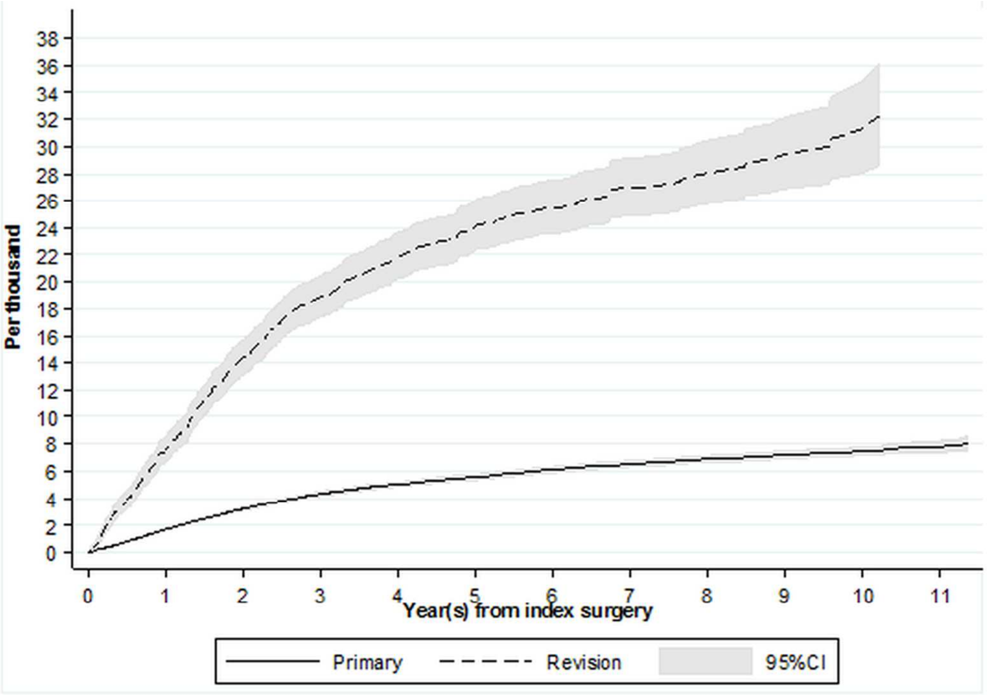

Figure 4
Figure 4
56x39mm (600 x 600 DPI)

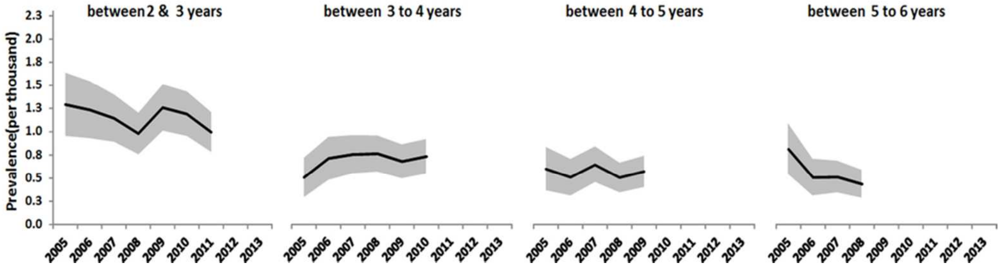

Web appendix Figure 1
Web appendix Figure1
40x10mm (600 x 600 DPI)

For peer review only

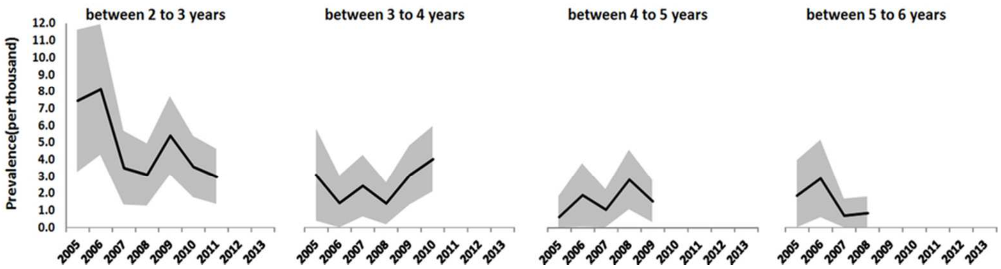

Web appendix Figure 2
Web appendix Figure 2
40x10mm (600 x 600 DPI)

For peer review only

Appendix Table 1: Characteristics of patients with a completed or partially-completed two-stage revision total knee replacement †

	Complete*	Only stage two**	Only stage one**
**Primary(n=2,833)**	1,478	792	563
Age(mean, SD)	66(9)	67(9)	67(10)
Female(%)	44	41	43
BMI(mean, SD)	32(6)	32(6)	32(6)
missing BMI (n=)	308	308	201
ASA 1(%)	14	14	10
ASA 2(%)	68	67	64
ASA 3(%)	18	19	25
ASA 4-5(%)	0	0	1
**Revision(n=576)**	294	154	128
Age(mean, SD)	66(10)	66(10)	69(10)
Female(%)	40	41	33
BMI(mean, SD)	32(6)	31(6)	32(6)
missing BMI	59	58	36
ASA 1(%)	14	10	13
ASA 2(%)	67	67	59
ASA 3(%)	19	22	27
ASA 4-5(%)	0	1	1

†2,833 two-stage revision total knee replacement procedures to manage an infection were recorded in the NJR following an index primary knee replacement and 576 following an index aseptic revision surgery.

* Both stage one and stage two procedures of a two-stage revision total knee replacement reported in the NJR.

**Only stage one or stage two procedure of a two-stage revision total knee replacement reported in the NJR.

For peer review only

STROBE 2007 (v4) checklist of items to be included in reports of observational studies in epidemiology*
Checklist for cohort, case-control, and cross-sectional studies (combined)

Section/Topic	Item #	Recommendation	Checked
**Title and abstract**	1	(*a*) Indicate the study's design with a commonly used term in the title or the abstract	**Page 1**
		(*b*) Provide in the abstract an informative and balanced summary of what was done and what was found	**Pages 2-3**
**Introduction**			
Background/rationale	2	Explain the scientific background and rationale for the investigation being reported	**Pages 5-6**
Objectives	3	State specific objectives, including any pre-specified hypotheses	**Page 6**
**Methods**			
Study design	4	Present key elements of study design early in the paper	**Page 7**
Setting	5	Describe the setting, locations, and relevant dates, including periods of recruitment, exposure, follow-up, and data collection	**Page 7+Figure 1**
Participants	6	Give the eligibility criteria, and the sources and methods of selection of participants. Describe methods of follow-up	**Page 7**
		For matched studies, give matching criteria and number of exposed and unexposed	na
Variables	7	Clearly define all outcomes, exposures, predictors, potential confounders, and effect modifiers. Give diagnostic criteria, if applicable	**Pages 7-8**
Data sources/ measurement	8*	For each variable of interest, give sources of data and details of methods of assessment (measurement). Describe comparability of assessment methods if there is more than one group	**Page 7**
Bias	9	Describe any efforts to address potential sources of bias	**Pages 7-8**
Study size	10	Explain how the study size was arrived at	**Page 7**
Quantitative variables	11	Explain how quantitative variables were handled in the analyses. If applicable, describe which groupings were chosen and why	**Pages 8-9**
Statistical methods	12	(*a*) Describe all statistical methods, including those used to control for confounding	**Pages 8-9**
		(*b*) Describe any methods used to examine subgroups and interactions	**Pages 8-9**
		(*c*) Explain how missing data were addressed	**Pages 7-8**
		(*d*) If applicable, explain how loss to follow-up was addressed	**Pages 8-9+Figure 1**
		(*e*) Describe any sensitivity analyses	na
**Results**			
Participants	13*	(a) Report numbers of individuals at each stage of study—eg numbers potentially eligible, examined for eligibility, confirmed eligible, included in the study, completing follow-up, and analysed	**Figure 1+Page 10**

For peer review only

		(b) Give reasons for non-participation at each stage	**Figure 1+Page 10**
		(c) Consider use of a flow diagram	**Figure 1+Page 10**
Descriptive data	14*	(a) Give characteristics of study participants (eg demographic, clinical, social) and information on exposures and potential confounders	**Page 10**
		(b) Indicate number of participants with missing data for each variable of interest	na
		(c)Summarise follow-up time (eg, average and total amount)	**Figures 2-4+Appendix figures 1-2+pages 10-13**
Outcome data	15*	Report numbers of outcome events or summary measures over time	**Tables 1-2+pages 10-13**
Main results	16	(*a*) Give unadjusted estimates and, if applicable, confounder-adjusted estimates and their precision (eg, 95% confidence interval). Make clear which confounders were adjusted for and why they were included	**Pages 10-13**
		(*b*) Report category boundaries when continuous variables were categorized	na
Other analyses	17	Report other analyses done—eg analyses of subgroups and interactions, and sensitivity analyses	**Pages 10-13**
**Discussion**			
Key results	18	Summarise key results with reference to study objectives	**Page 14**
Limitations	19	Discuss limitations of the study, taking into account sources of potential bias or imprecision. Discuss both direction and magnitude of any potential bias	**Page 15**
Interpretation	20	Give a cautious overall interpretation of results considering objectives, limitations, multiplicity of analyses, results from similar studies, and other relevant evidence	**Pages 16-17**
Generalisability	21	Discuss the generalisability (external validity) of the study results	**Pages 15-16**
**Other information**			
Funding	22	Give the source of funding and the role of the funders for the present study and, if applicable, for the original study on which the present article is based	**Page 18**

*Give information separately for cases and controls in case-control studies and, if applicable, for exposed and unexposed groups in cohort and cross-sectional studies.

Note: An Explanation and Elaboration article discusses each checklist item and gives methodological background and published examples of transparent reporting. The STROBE checklist is best used in conjunction with this article (freely available on the Web sites of PLoS Medicine at http://www.plosmedicine.org/, Annals of Internal Medicine at http://www.annals.org/, and Epidemiology at http://www.epidem.com/). Information on the STROBE Initiative is available at www.strobe-statement.org.

For peer review only

Description of the rates, trends and surgical burden associated with revision for prosthetic joint infection following primary and revision knee replacement in England and Wales. An analysis of the National Joint Registry for England, Wales, Northern Ireland and the Isle of Man.

Journal:	*BMJ Open*
Manuscript ID	bmjopen-2016-014056.R1
Article Type:	Research
Date Submitted by the Author:	24-Feb-2017

Complete List of Authors:	Lenguerrand, Erik; University of Bristol School of Clinical Science, School of Clinical Sciences, Musculoskeletal Research Unit<br>Whitehouse, Michael; University of Bristol, Musculoskeletal Research Unit<br>Beswick, Andrew; University of Bristol, School of Clinical Sciences<br>Toms, Andrew ; Royal Devon and Exeter NHS trust<br>Porter, Martyn; Wrightington Hospital<br>Blom, Ashley; University of Bristol, School of Clinical Sciences

**Primary Subject Heading**:	Surgery
Secondary Subject Heading:	Epidemiology
Keywords:	Knee < ORTHOPAEDIC & TRAUMA SURGERY, Primary knee replacement, Revision knee replacement, prosthetic joint infection, Registry

SCHOLARONE™ Manuscripts

For peer review only

Description of the rates, trends and surgical burden associated with revision for prosthetic joint infection following primary and revision knee replacement in England and Wales. An analysis of the National Joint Registry for England, Wales, Northern Ireland and the Isle of Man.

*Erik Lenguerrand[1], *Michael R Whitehouse[1], Andrew D Beswick[1], Andrew D Toms[2], Martyn L Porter[3], Ashley W Blom[3], on behalf of the National Joint Registry for England, Wales, Northern Ireland and the Isle of Man.

* Authors contributed equally to this work

1. Musculoskeletal Research Unit, School of Clinical Sciences, University of Bristol, Bristol, UK
2. Exeter Knee Reconstruction Unit, Princess Elizabeth Orthopaedic Centre, Royal Devon and Exeter NHS trust, Exeter, UK
3. Centre for Hip Surgery, Wrightington Hospital, Lancashire, WN6 9EP

Correspondence to:
Name: Dr Erik Lenguerrand
Address: Musculoskeletal Research Unit, 1st Floor Learning & Research Building, School of Clinical Sciences, University of Bristol, Southmead Hospital, Bristol, BS10 5NB, UK
Email: erik.lenguerrand@bristol.ac.uk Tel: 0117 414 7865 Fax: 0117 414 7924

Corresponding author:
Name: Prof Ashley W Blom
Address: Musculoskeletal Research Unit, 1st Floor Learning & Research Building, School of Clinical Sciences, University of Bristol, Southmead Hospital, Bristol, BS10 5NB, UK
Email: ashley.blom@nbt.nhs.uk Tel: 0117 414 7865

Co-corresponding author:
Name: Mr Martyn L Porter
Address: Centre for Hip Surgery, Wrightington Hospital, Lancashire, WN6 9EP, UK
Email: martynporter@hipkneeclinic.com Tel: 01257 266 166

For peer review only

Word count: 4152

ABSTRACT

Objectives

To describe the prevalence rates of revision surgery for the treatment of prosthetic joint infection (PJI) for patients undergoing knee replacement, their time trends, the cumulative incidence function of revision for PJI and estimate the burden of PJI at health service level.

Design

We analysed revision knee replacements performed due to a diagnosis of PJI and the linked index procedures recorded in the National Joint Registry from 2003–2014 for England and Wales. The cohort analysed consisted of 679,010 index primary knee replacements, 33,920 index revision knee replacements and 8,247 revision total knee replacements performed due to a diagnosis of PJI. The prevalence rates, their time-trends investigated by time from index surgery to revision for PJI, cumulative incidence functions, and the burden of PJI (total procedures) were calculated. Overall linear trends were investigated with log-linear regression.

Results

The incidence of revision total knee replacement due to PJI at two years was 3.2/1,000 following primary and 14.4/1,000 following revision knee replacement respectively. The prevalence of revision due to PJI in the 3 months following primary knee replacement has risen by 2.5-fold (95%CI 1·2–5·3) from 2005–2013 and 7.5-fold (95%CI 1·0–56.1) following revision knee replacement. Over 1,000 procedures/year are performed as a consequence of knee PJI, an increase of 2.8 from 2005-2013. Overall, 75% of revisions were two-stage with an increase in use of single stage from 7.9% in 2005 to 18.8% in 2014.

Conclusions

For peer review only

Although the risk of revision due to PJI following knee replacement is low, it is rising, and coupled with the established and further predicted increased incidence of both primary and revision knee replacement, this represents an increasing and substantial treatment burden for orthopaedic service delivery in England and Wales. This has implications for future service design and the funding of individual and specialist centres.

Keywords: Orthopaedics, Primary knee replacement, Revision knee replacement, prosthetic joint infection, Registry

For peer review only

For peer review only - http://bmjopen.bmj.com/site/about/guidelines.xhtml

STRENGHT AND LIMITATIONS OF THIS STUDY

- This study provides updated and nationally representative descriptive evidence for England and Wales on revision for prosthetic joint infection following knee replacements.
- Over 600,000 index primary replacements, 30,000 index revision replacements and 8,000 revision total replacements performed due to a diagnosis of infection are included in the analyses.
- Contemporary evidence regarding changes over time in the risk of revision for prosthetic joint infection, its burden, and the types of revision surgery used for managing prosthetic joint infection is provided.
- The diagnosis of prosthetic joint infection recorded in the registry is not validated against a recognised international standard and may therefore be subject to over or under reporting.
- Not all recorded revisions for prosthetic joint infection had an index primary or aseptic revision total knee replacement recorded in the National Joint Registry.

For peer review only

BACKGROUND

Deep prosthetic joint infection (PJI) is a rare but serious complication of total knee replacement. Patients experience severe pain, functional difficulties, poor quality of life and, if untreated, loss of the affected limb or death. [1-3]

Estimates of the incidence of PJI after primary knee replacement range between 0.85% in Germany, 1.0% in the UK, 1.4% in Finland and 2.2% in the USA.[4-7] In a study from Finland, about 1.14% of patients had a PJI within 2 years of their primary knee replacement with a further 0.27% of patients presenting with PJI later than 2 years after primary surgery.[6] After aseptic revision and more complex knee replacement surgeries, infection rates may be considerably higher.[8] There is a paucity of evidences from UK centres and the available studies are old and based on single-centre data.[4 9]

Bacteria adhere as biofilm to implants and peri-prosthetic tissues making the treatment of PJI difficult.[10] Patients with PJI after knee replacement and their treating surgeons face complex and protracted treatment pathways. Initial treatment may involve surgical debridement, antibiotics and implant retention (DAIR), particularly in early postoperative infections. However about 45-52% of patients receiving DAIR may subsequently need revision of their implants.[11] Rates of revision of implants following treatment with a DAIR may be lower with strict selection criteria [12], but larger single centre cohort studies suggest the rate remains around 20% by two years [13].Delays to effective infection control inherent in this process may lead to a poor post replacement outcome.[14]

For patients with PJI after knee replacement who are diagnosed before biofilm formation occurs, DAIR with modular exchange is a reasonable treatment option but for the majority of patients outside of this window of opportunity, contemporary management involves surgical revision with extensive debridement and antibiotic treatment with either immediate implant replacement (single-stage) or delayed implant replacement (two-stage).

The two-stage revision procedure was the first method reported for treatment of PJI of the knee that aimed to restore a functioning, painless knee.[15] The period between operations allows targeted antibiotic treatment and monitoring of clinical status and inflammatory markers. However, patients require two distinct planned major surgeries and a period of several months with limited knee function and protracted distress, concern and uncertainty.[16] An alternate one-stage strategy was developed by the Endo-Klinik and its use reported from 1976.[17] Key to the single-stage revision strategy is

For peer review only

extensive debridement, identification of the infecting organisms and appropriate tailored antibiotic treatment.[18]

There has been no randomised trial comparing the outcome of single-stage and two-stage revision for PJI of the knee but evidence from case-series suggests similar rates of re-infection after each treatment.[19] The decision to treat with a single-stage or two-stage procedure may be guided by microbiological tests and patient health but ultimately the choice of revision method is largely that of the treating surgeon. [2]

The National Joint Registry for England, Wales Northern Ireland and the Isle of Man (NJR) is the largest joint replacement database in the world and has collected data since April 2003.[20]

We analysed this dataset to assess the burden of revision surgery for knee PJI between April 2003 and December 2013. In this period, data was available from England and Wales. Our specific aims were to:

- Present the surgeon's perspective by describing the prevalence rates of revision surgery for the treatment of knee PJI following primary and revision surgery for aseptic indications, and their time trends broken down by time from index surgery to revision for infection.
- Present the patient's perspective by deriving cumulative incidence functions by type of index surgery.
- Estimate the burden of revision surgery for knee PJI at a health service level by accounting for all of the registered revisions and re-revision surgeries performed for knee PJI.

For peer review only

METHODS

Data source

In this observational study, we report analyses of data from the NJR.[20] The registry was established in 2003 and includes details of primary and revision knee replacements performed in England and Wales. Data entry for Northern Ireland and the Isle of Man did not commence until 2013 and 2015 respectively and data linkage for those periods is limited, therefore they are excluded from this analysis.

Index surgeries and revision surgeries for PJI

We grouped procedures as index knee replacements and revision surgeries for treatment of knee PJI. Index surgeries included all primary procedures and all revision procedures performed for an indication other than infection. The index revision procedures have been labelled "aseptic revision" procedures to indicate they were not performed due to PJI. All index surgeries performed between 1st April 2003 and 31st December 2013 were included (to allow a minimum 12-months follow-up).

Information on microbiology results are not recorded in the NJR and infected index procedures were identified using subsequent revision performed for an indication of PJI. The diagnosis and treatment strategy for PJI is at the discretion of the surgeon according to local protocols and available information at the time of surgery. Revision surgeries performed as a consequence of PJI between 1st April 2003 and 31st December 2014 were considered. Revisions not performed for PJI but that were performed on a knee previously operated on due to PJI were not considered as index procedures, but were used alongside the revision and re-revision procedures for PJI to define the "burden of PJI". Non-surgical management of PJI and surgical procedures where no revision or modular exchange of implants is performed are not recorded in the NJR.

Revision procedures are reported in the NJR as a single stage, a stage one of a two-stage revision, a stage two of a two-stage revision procedure, a conversion to arthrodesis, or an amputation. DAIR procedures with modular exchange are recorded in the NJR dataset as single stage revision procedures. To identify which procedures recorded as single stage revisions were DAIRs with modular exchange as opposed to complete single stage revisions where implants fixed to bone are also revised, the component level data was considered for the index and revision procedures. Implant component labels are compulsory for all NJR records, the minimum dataset forms also contain fields for

For peer review only - http://bmjopen.bmj.com/site/about/guidelines.xhtml

components removed but this information is discretionary and limited to the brand removed, therefore does not allow component tracing unless a linked index procedure exists. Procedures were therefore defined on the basis of the data provided for components implanted at revision surgery. Procedures recorded as single stage revisions where only modular components were added (termed "meniscal component" in the minimum data set) were defined as DAIRs with modular exchange. Those where implants fixed to bone were implanted ("femoral component", "tibial tray" +/- "patella") were defined as single stage revisions. Debridements where the surgeon either elects to not exchange modular components when they are present or where modular exchange is not possible (e.g. with monoblock polyethylene tibial components) are not captured in the NJR.

Missing procedures

There were 2,880 two-stage revision procedures recorded where the index surgery was a primary knee replacement, in 792 (27%) of these, only a stage two of a two-stage procedure was recorded in the NJR. This was also observed for 154 (26%) of the 589 two-stage revision procedures following an index aseptic revision procedure. Patients with incompletely registered two-stage procedures did not differ from those with complete information in terms of gender, BMI, and ASA grade (see appendix Table 1). For index primary knee replacement, patients with incomplete information were on average one year older than those with complete information (p=0.05). For aseptic revision surgeries, no age difference was observed between the two groups.

For incomplete procedures, the date of the stage one of a two-stage revision procedure was estimated. Firstly we derived the relative weight of time elapsed between the index and stage one of a two-stage procedure by year and type of index surgery using patients with complete information: 100x(length of time$_{\text{index surgery- 1st stage}}$/length of time $_{\text{index surgery- 2nd stage}}$). We applied these weights to the length of time observed between the index and stage two of a two-stage procedure for those with incomplete information to obtain an estimate for the time between the index surgery and stage one of the revision surgery.

Statistical analyses

Analyses were performed with Stata SE 13.1 (StataCorp LP, TX, USA).

For peer review only - http://bmjopen.bmj.com/site/about/guidelines.xhtml

Surgeon perspective

Prevalence rates of index surgeries performed between 2003–2013 and subsequently revised for infection between 2003–2014 were derived by year and type of index surgery. This provides a “surgeon” perspective of revision for PJI by describing the proportion of knee replacements which required revision surgery for the management of infection. The prevalence rates were plotted by time from index surgery to revision for infection (≤3 months, 3–6 months, 6 months–1 year, 1–2 years, 2–3 years, 3–4 years, and 5–6 years). For two-stage surgeries, the date of stage one of a two-stage revision was selected to indicate the date of revision for PJI. Log-linear regression, using the year of the index knee replacement as a continuous independent factor, was used to investigate overall linear trends between 2005 and 2013. This period was selected as over 85% (proportion of procedure records submitted to the NJR compared with the levy returns for the number of implants sold) of knee replacements performed in 2005 and over 99% of those performed from 2007 onwards had been recorded in the NJR; prior to 2005, the data capture of the NJR was <75%.[21] When evidence of a time-trend was identified (year of surgery, $p \leq 0.05$), the year of surgery was re-considered as a categorical variable using 2005 as the reference period. Estimated relative risk and related 95% confidence intervals (95%CI) quantified the relative increase in prevalence rates between the period of interest and 2005.

Patient perspective

To move from a surgical perspective to a patient perspective, cumulative incidence functions [22-24] were derived by type of index surgery (primary or aseptic revision). These provide the probability of being revised as a consequence of PJI within a specific time period following the index surgery while accounting for the time patients were at risk of being revised for a PJI and the competing risks of death and revision for an aseptic indication.

For peer review only - http://bmjopen.bmj.com/site/about/guidelines.xhtml

For peer review only

Healthcare service perspective

The overall "burden of revision surgery for knee PJI" was analysed using all revision procedures performed between 2003 and 2014 for an indication of PJI as well as any subsequent re-revision procedures for PJI or another indication.

Typically, knee replacement survivorship is 10 years or more following surgery,[20] re-revision procedures performed for another indication than PJI during the observation period are therefore considered as a consequence of the earlier management of PJI.

Revision procedures for an indication of PJI with no index procedure recorded in the NJR (n=3,064, Figure 1) and their re-revision procedures were also accounted. Contrary to the previous analyses in which index surgeries performed in 2014 and their revisions procedures were not considered to allow for a minimum of one-year post-operative follow-up, revisions for PJI relating to index knee replacements performed in 2014 (n=122) were included in this analysis.

The overall burden was reported by the year and type of revision surgery. Stage one of a two-stage revision and stage two of a two-stage revision were considered as one procedure to avoid double counting. The same strategy was also used to account for multiple stage ones of a two-stage revision procedure.

For peer review only

RESULTS

Surgeon perspective

There were 679,010 primary and 33,920 aseptic revision knee replacements recorded in the NJR for England and Wales between April 2003 and December 2013 (Table 1).

A total of 3,659 patients required at least one revision surgery due to PJI (4,004 procedures) following a primary knee replacement (Figure 1.A). A further 717 patients required a subsequent revision for PJI (785 procedures) following an aseptic revision knee replacement (Figure 1.B). For 2,918 patients who underwent revision for PJI (3,187 procedures), no index surgery was recorded(Figure 1.C).

<Figure 1 here>

Figure 1. Description of procedures recorded in the National Joint Registry.

Of the primary knee replacements recorded in the NJR, 5.39/1,000 (95%CI 5.21-5.56) were subsequently revised due to PJI (Table 1). Of the aseptic revision knee replacements, 21.14/1,000(95%CI 19.61-22.67) were subsequently revised due to PJI.

For peer review only

Table 1. Primary and aseptic revision knee replacement procedures revised for a prosthetic knee joint infection (PJI).

Year of index procedure	Index primary replacement			Index aseptic revision replacement		
	Total N=	Revised for PJI n=	Prevalence rate Per 1,000, 95%CI	Total N=	Revised for PJI n=	Prevalence rate Per 1,000, 95%CI
**Total**	**679,010**	**3,659**	**5.39(5.21, 5.56)**	**33,920**	**717**	**21.14(19.61, 22.67)**
2003	13,547	112	8.27(6.74, 9.79)	534	24	44.94(27.37, 62.52)
2004	27,706	184	6.64(5.68, 7.60)	1,001	24	23.98(14.50, 33.45)
2005	41,848	314	7.50(6.68, 8.33)	1,612	41	25.43(17.75, 33.12)
2006	49,482	346	6.99(6.26, 7.73)	2,094	55	26.27(19.42, 33.12)
2007	66,618	471	7.07(6.43, 7.71)	2,847	60	21.07(15.80, 26.35)
2008	73,859	443	6.00(5.44, 6.55)	3,538	87	24.59(19.49, 29.69)
2009	75,612	465	6.15(5.59, 6.71)	3,884	91	23.43(18.67, 28.19)
2010	78,276	423	5.40(4.89, 5.92)	4,196	102	24.31(19.65, 28.97)
2011	81,795	378	4.62(4.16, 5.09)	4,336	81	18.68(14.65, 22.71)
2012	85,161	341	4.00(3.58, 4.43)	5,115	93	18.18(14.52, 21.84)
2013	85,106	182	2.14(1.83, 2.45)	4,763	59	12.39(9.25, 15.53)

For peer review only

For peer review only - http://bmjopen.bmj.com/site/about/guidelines.xhtml

Figure 2 shows trends in revision for PJI in the 2 years after primary knee replacement. Revision rates within 3 months of the index procedure increased over time, with the prevalence rate in 2013 over twice that in 2005, RR 246 (95%CI 1.15-5.25; time trend p<0.0001). No time-trends for revision rates between 2005 and 2013 were apparent in the periods 3–6 months, 6 months–1 year and 1–2 years. Other than the prevalence rate of revision for PJI between 5–6 years which decreased over time, RR 0·53 (95%CI 0.33-0.86; time trend p=0.018), there was no evidence of time-trends for the rates of late revision for PJI (Web appendix Figure1).

<Figure 2 here>

Figure 2. Prevalence (95%CI) of revision for prosthetic joint infection within 2 years of the index primary knee replacement.

Trends in subsequent revision of aseptic revision knee replacements for PJI are shown in Figure 3 and Web appendix Figure 2. The prevalence of aseptic revisions revised for PJI within 3 months of the procedure increased over time, RR 7.47 (95%CI 1.00-56.12; time trend p=0.001 for 2013 compared to 2006). No important differences were noted in the prevalence rates of revision for PJI performed at any time beyond 3 months from the index revision surgery (all time trend p-values >0.1) with the exception of revision for infection between 2–3 years for which the rates decreased over time, RR 0.40 (95%CI 0.18-0.88; time trend p=0.008 for 2011 compared to 2005).

<Figure 3 here>

Figure 3. Prevalence (95%CI) of revision for prosthetic joint infection within 2 years of the index aseptic revision knee replacement.

Patient perspective

Figure 4 shows the incidence over time of revision for PJI in patients with an index primary or aseptic revision while accounting for the risk of death and revision for any indication other than PJI. The probability of revision for PJI at one year following a primary knee replacement 1.7/1,000 (95%CI 1.6-1.8) compared to 7.6/1,000 (95%CI 6.8-8.6) following an aseptic revision knee replacement. At two years the probability was 3.2/1,000 (95%CI 3.1-3.4) and 14.4/1,000(95%CI 13.1-15.7) respectively, and at five years it was 5.6/1,000 (95%CI 5.4-5.8) and 24.1/1,000 (95%CI 22.3-26.1) respectively. The

For peer review only

probability of revision for infection within the first ten years following primary knee replacement was 7.5/1,000 (95%CI 7.2-7.8) and 31.3/1,000 (95%CI 28.1-24.9) following an aseptic revision.

<Figure 4 here>

Figure 4. Cumulative incidence function of revision for prosthetic joint infection following index primary and aseptic revision knee replacement.

Healthcare service perspective

Table 2 summarises the revision surgeries for the management of PJI and subsequent re-revisions (including repeated procedures to manage PJI and other revision procedures, see Figure 1 (A-C)) performed after primary and aseptic revision knee replacement by the year the (re-)revision was performed.

The absolute number of procedures performed as a consequence of PJI in England and Wales has increased from 378 in 2005 to 1,048 in 2014, a relative increase of 2.8 fold. This is higher than the 2.1 fold increase in primary knee replacements over the same period, but similar to the 2.9 increase in aseptic revision surgeries between 2005 and 2013 (table 1).

Overall, 75% of revisions were conducted with a two-stage procedure but the use of single-stage revision for PJI has increased from 7.9% in 2005 to 18.8% in 2014. The median interval between stages in a two-stage revision following a primary index surgery was 99 days (25th-75th percentiles: 68, 156). A 101 day (67, 147) median interval was observed for two-stage revision performed to manage PJI following an index aseptic revision surgery. The DAIR procedures represented around 5% of the total reported procedures and 9% of those reported in 2014. Other types of revision surgeries were rare and consisted mainly of arthrodesis (110 out of 134).

For peer review only

Table 2. Revision procedures performed as a consequence of prosthetic joint infection (PJI) by type and year of procedure *

Year of revision procedure	Total N=	DAIR n=, (%)	Single stage n=, (%)	Two-stage n=, (%)	Other† n=, (%)
**Total**	**8,587**	408(4.8)	1,589(18.5)	**6,456**(75.2)	**134**(1.6)
2003	126	3(2.4)	116(92.1)	7(05.6)	0(0.0)
2004	236	3(1.3)	59(25.0)	171(72.5)	3(1.3)
2005	378	8(2.1)	30(07.9)	336(88.9)	4(1.1)
2006	485	11(2.3)	49(10.1)	419(86.4)	6(1.2)
2007	663	19(2.9)	70(10.6)	560(84.5)	14(2.1)
2008	783	29(3.7)	124(15.8)	615(78.5)	15(1.9)
2009	852	32(3.8)	145(17.0)	662(77.7)	13(1.5)
2010	931	38(4.1)	178(19.1)	707(75.9)	8(0.9)
2011	1,004	51(5.1)	187(18.6)	748(74.5)	18(1.8)
2012	1,035	58(5.6)	200(19.3)	754(72.9)	23(2.2)
2013	1,046	61(5.8)	234(22.4)	732(70.0)	19(1.8)
2014	1,048	95(9.1)	197(18.8)	745(71.1)	11(1.1)

*This table reports the revision procedures performed after any index surgery revised as a consequence of PJI including subsequent re-revision procedures whether performed to manage an infection or not. It also includes the revision procedures performed between 2003 and 2014 on 3,064 patients with prosthetic joint infection but with no index procedure documented in the NJR.
† Conversion to arthrodesis or amputation

DISCUSSION

This study is the largest to date investigating the treatment of PJI after knee replacement. It includes 712,930 primary and aseptic revision index procedures and 8,247 revision total knee replacements performed due to a diagnosis of PJI. We have shown that the prevalence and incidence rates of revision for PJI are higher following aseptic revision knee replacement than for primary knee replacement and that the prevalence of revision for PJI within 3 months of surgery has risen over time for primary and aseptic revision procedures. The total burden of treating PJI of the knee has risen substantially, with over 1,000 procedures performed per year from 2011 onwards, which mirrors the greater rise in aseptic revision surgery compared to primary knee replacements.[20] From 2005, we have identified an increased use of single stage as compared to two-stage revision for PJI of the knee which appears to have plateaued at approximately 20%.

For peer review only - http://bmjopen.bmj.com/site/about/guidelines.xhtml

Strengths and weaknesses

The NJR is an established, large, prospective, observational arthroplasty register with comprehensive coverage of procedures undertaken, which is reassuring in terms of the generalisability of the data generated by the study. The data has some limitations. The recording of PJI as an indication for surgery is according to the opinion of the treating surgeon at the time of surgery and, as such, the diagnosis has not necessarily been referenced against a gold standard set of criteria and hence may be subject to misdiagnosis. Whilst the NJR now represents a mature dataset with long term follow up, there are still a substantial number of procedures undergoing revision knee replacement where there is no record of a linked index primary or revision knee replacement or where there are an incomplete set of procedures recorded for two stage revision surgery. This may be a reflection of the high survivorship of knee replacement for 10 years or more following surgery,[20] the fact that some PJI is acquired late and that not every case performed since 2003 in England and Wales has been recorded in the NJR. Given there was no difference between patients with complete two-stage episodes recorded and those with partial episodes recorded in terms of age, gender, BMI and ASA grade, we have assumed that the time interval of the former is generalisable to the latter, which may not be the case. The NJR does not capture information on microbiology results, non-surgical treatment of PJI and revision for PJI with implant retention but no modular exchange. Currently in the NJR, the only option to record DAIR procedures where modular exchange is performed is to record them as single stage revision surgeries.[25] This complicates their differentiation from single stage surgeries where the femoral and tibial components are revised. This represents a potential weakness of the data collection form as this may be subject to different interpretation by surgeons, despite the fact that recording of procedures in which any component is removed or inserted is mandatory. We have used component level data for individual cases to identify DAIR procedures with modular exchange. A few single-stage revisions for PJI unlinked to an index procedure were considered as DAIRs in view of the components implanted (n=66). The capture of revision surgeries, in particular those performed for PJI is also not perfect in arthroplasty registries [26-29] and it is unclear how complete the capture of these procedures in the NJR is.

The annual burden of PJI with over 1,000 procedures in recent years, while already high and expensive, is a conservative estimation and the cost of PJI considerable for the NHS.

For peer review only - http://bmjopen.bmj.com/site/about/guidelines.xhtml

Comparison with other studies

Revision for infection is a rare complication of knee replacement. Our findings in this respect are consistent with previous studies from England and Wales [4 9] although the reported prevalence rates were higher in previous studies (around 1% at 5 years following primary replacement compared to 0.5% in our study). Our study was not contemporaneous with those studies (index surgeries performed between 1987-2001 [9] or 1993-1996 [4]) and the considered sample size in them was smaller (n=4,788 and n=931). Evidence from other countries shows similar prevalence rates of revision for infection (rates ranging from 0.4% to 2.2%).[5-7 30 31] Studies which report incidence rates typically include any type of operations for the management of infection (not just those where implants are removed or changed) and this may be why they show higher rates (0.9% to 1.4% between 1 and 5 years postoperatively).[6 32]

We have demonstrated a substantial time trend of an increased risk of revision for PJI within the first 3 months of an index primary or aseptic revision knee replacement being performed. This phenomenon is likely to be multifactorial. Factors that could lead to an increased diagnosis of PJI in this period and hence increased risk of revision include the increased accuracy of tests available to clinicians for the diagnosis of PJI,[33] coupled with more rapid diagnosis and/or treatment in specialist centres, the increased risk factors for PJI amongst the population undergoing knee replacement population (such as elevated body mass index [34]) and increased bed occupancy within the healthcare setting in which these procedures were performed.[35] There may be a trend towards the use of revision surgery to manage PJI rather than suppressive treatment with antibiotics but we cannot comment on this as non-surgical management of PJI is beyond the scope of this study. There have been similar findings in other countries that could not be accounted for by risk factors recorded in those registries, suggesting this trend could reflect an actual general increase in the risk of PJI. [36] It is interesting to note from our results that there has not been a relative increase in the use of DAIRs with modular exchanges in the NJR to explain this phenomenon.

We have shown a greater incidence of revision for PJI following aseptic revision knee replacement (3.1% at 10 years) compared to primary knee replacement (0.8% at 10 years) consistent with the findings of previous cohort studies.[4 31 32 37-39] Previous surgery is a known risk factor for PJI, [40]

For peer review only - http://bmjopen.bmj.com/site/about/guidelines.xhtml

this may because of further bacterial contamination or increased length of surgery. Revision knee replacement tends to be performed in a population with increased host [41] and procedure risk factors for infection or further revision [42] and involve the introduction of a greater volume of prosthetic material and additional adjuncts such as bone graft that may present a favourable environment for bacterial colonisation and subsequent PJI. [43]

Implications for clinicians and policymakers

The current rate of surgical revision of PJI of the knee stands at approximately 1,000 cases per year across the NJR. This represents a significant healthcare burden which has more than doubled over the last decade, exceeding the rate of increase in primary knee replacements. The cost of revision knee replacement due to PJI is more than three times that of primary knee replacement and over twice that of aseptic revision.[44] In the National Health Service, the costs associated with revision knee replacement due to PJI have been shown to be in excess of £30,000 per case, greater than other indications for revision,[45] even before accounting for costs associated with litigation. Single stage revision offers an advantage over the two-stage approach in terms of both patient and surgeon derived utility values in both the short and long term in the hip [46] and may be associated with superior functional outcomes in the knee [47] suggesting this increased burden could be ameliorated by the increased use of a single stage revision strategy.

Unanswered questions and future research

The observed trend of an increase in the risk of infection in the first 3 months following primary and aseptic revision total knee replacement is not currently well understood. Identification of the risk factors associated with this phenomenon and modifiable risk factors for PJI may help to reduce this risk. Although the utilisation of single stage revision knee replacement for the treatment of PJI has increased to approximately 20%, given the current evidence base shows equivalent reinfection rates following single stage compared to two-stage revision surgery for PJI,[19 48] it might be possible that the proportion of patients treated with single-stage surgery taking account of patient, surgeon, and operative factors could be further increased. Given the increasing burden of treating PJI, this may help ameliorate the increase in resources that will otherwise be required to treat this condition [49]. There

For peer review only

is currently no data available from randomised controlled trials looking at the outcome of these two treatment strategies and such evidence is required to support the decision making process in the planning and treatment of these cases.

For peer review only

DECLARATIONS

Acknowledgments

We thank the patients and staff of all the hospitals who have contributed data to the National Joint Registry. We are grateful to the Healthcare Quality Improvement Partnership, the National Joint Registry Steering Committee, and staff at the National Joint Registry for facilitating this work.

Competing interest

MP is the Medical Director of the National Joint Registry and also acts as Chair of the Programme Steering Committee for the National Institute for Health Research (NIHR) INFORM program (PGfAR program: RP-PG-1210-12005). The other authors have no conflicts of interest.

Funding statement

This article presents independent research funded by the National Institute for Health Research (NIHR) under its Programme Grants for Applied Research program (RP-PG-1210-12005). The funders had no role in study design, data collection and analysis, decision to publish, or preparation of the manuscript.

Authors' contributions

EL, MRW, ADB and AWB designed the study. The data were extracted by Northgate (Hemel Hempstead, UK). EL, ADB, MRW, and AWB reviewed the published work. EL conducted the statistical analyses. All authors interpreted data and wrote the report. EL had full access to all the data and AWB is the guarantor.

Disclaimer

The views expressed represent those of the authors and do not necessarily reflect those of the National Joint Registry Steering Committee or Healthcare Quality Improvement Partnership, who do not vouch for how the information is presented.

For peer review only

The views expressed in this article are those of the authors and not necessarily those of the NHS, the NIHR, or the Department of Health.

Ethic approval and consent to participate

Patient consent was obtained for data collection by the National Joint Registry. According to the specifications of the NHS Health Research Authority, separate informed consent and ethical approval were not required for the present study.

Availability of data and materials

Access to the data analysed in this study required permission from the National Joint Registry for England, Wales and Northern Ireland Research Sub-committee. http://www.njrcentre.org.uk/njrcentre/Research/Researchrequests/tabid/305/Default.aspx contains information on research data access request to the National Joint Registry.

For peer review only

References

1. Cahill JL, Shadbolt B, Scarvell JM, et al. Quality of life after infection in total joint replacement. J Orthop Surg 2008;**16**(1):58-65.

2. Gehrke T, Alijanipour P, Parvizi J. The management of an infected total knee arthroplasty. Bone Joint J 2015;**97-B**(10 Suppl A):20-9, doi: 10.1302/0301-620X.97B10.36475.

3. Zmistowski B, Karam JA, Durinka JB, et al. Periprosthetic joint infection increases the risk of one-year mortality. J. Bone Joint Surg. Am. 2013;**95**(24):2177-84, doi: http://dx.doi.org/10.2106/JBJS.L.00789.

4. Blom AW, Brown J, Taylor AH, et al. Infection after total knee arthroplasty. J. Bone Joint Surg. Br. 2004;**86-B**(5):688-91.

5. Meyer E, Weitzel-Kage D, Sohr D, et al. Impact of department volume on surgical site infections following arthroscopy, knee replacement or hip replacement. BMJ Qual Saf 2011;**20**(12):1069-74, doi: 10.1136/bmjqs.2011.051250.

6. Huotari K, Peltola M, Jamsen E. The incidence of late prosthetic joint infections: a registry-based study of 112,708 primary hip and knee replacements. Acta Orthop. 2015;**86**(3):321-5, doi: 10.3109/17453674.2015.1035173.

7. Kurtz SM, Lau E, Watson H, et al. Economic burden of periprosthetic joint infection in the United States. J. Arthroplasty 2012;**27**(8 Suppl):61-5, doi: 10.1016/j.arth.2012.02.022.

8. Mortazavi SM, Schwartzenberger J, Austin MS, et al. Revision total knee arthroplasty infection: incidence and predictors. Clin. Orthop. Relat. Res. 2010;**468**(8):2052-9, doi: 10.1007/s11999-010-1308-6.

9. Phillips JE, Crane TP, Noy M, et al. The incidence of deep prosthetic infections in a specialist orthopaedic hospital: a 15-year prospective survey. J. Bone Joint Surg. Br. 2006;**88**(7):943-8.

10. Arnold WV, Shirtliff ME, Stoodley P. Bacterial biofilms and periprosthetic infections. J. Bone Joint Surg. Am. 2013;**95**(24):2223-9.

11. Romano CL, Manzi G, Logoluso N, et al. Value of debridement and irrigation for the treatment of peri-prosthetic infections. A systematic review. Hip Int. 2012;**22 Suppl 8**:S19-24, doi: http://dx.doi.org/10.5301/HIP.2012.9566.

12. Laffer RR, Graber P, Ochsner PE, et al. Outcome of prosthetic knee-associated infection: evaluation of 40 consecutive episodes at a single centre. Clin. Microbiol. Infect. 2006;**12**(5):433-9.

For peer review only

13. Byren I, Bejon P, Atkins BL, et al. One hundred and twelve infected arthroplasties treated with 'DAIR' (debridement, antibiotics and implant retention): antibiotic duration and outcome. J. Antimicrob. Chemother. 2009;**63**(6):1264-71, doi: 10.1093/jac/dkp107.

14. Sherrell JC, Fehring TK, Odum S, et al. Fate of two-stage reimplantation after failed irrigation and debridement for periprosthetic knee infection. Clin. Orthop. Relat. Res. 2011;**469**(1):18-25.

15. Insall JN, Thompson FM, Brause BD. Two-stage reimplantation for the salvage of infected total knee arthroplasty. J. Bone Joint Surg. Am. 1983;**65**(8):1087-98.

16. Moore AJ, Blom AW, Whitehouse MR, et al. Deep prosthetic joint infection: a qualitative study of the impact on patients and their experiences of revision surgery. BMJ Open 2015;**5**(12):e009495, doi: 10.1136/bmjopen-2015-009495.

17. von Foerster G, Kluber D, Kabler U. Mid- to long-term results after treatment of 118 cases of periprosthetic infections after knee joint replacement using one-stage exchange surgery. Orthopade 1991;**20**(3):244-52.

18. Tibrewal S, Malagelada F, Jeyaseelan L, et al. Single-stage revision for the infected total knee replacement: results from a single centre. Bone Joint J 2014;**96-B**(6):759-64, doi: 10.1302/0301-620X.96B6.33086.

19. Kunutsor SK, Whitehouse MR, Lenguerrand E, et al. Re-Infection Outcomes Following One- And Two-Stage Surgical Revision of Infected Knee Prosthesis: A Systematic Review and Meta-Analysis. PLoS One 2016;**11**(3):e0151537, doi: 10.1371/journal.pone.0151537.

20. *National Joint Registry for England, Wales and Northern Ireland: 12th Annual Report*. Hemel Hempstead: NJR Centre 2015.

21. *National Joint Registry for England and Wales: 9th Annual Report.* Hemel Hempstead: NJR Centre, 2012.

22. Coviello V, M. B. Cumulative incidence estimation in the presence of competing risks. Stata Journal 2004(4):103–12.

23. Gillam MH, Ryan P, Graves SE, et al. Competing risks survival analysis applied to data from the Australian Orthopaedic Association National Joint Replacement Registry. Acta Orthop. 2010;**81**(5):548-55, doi: 10.3109/17453674.2010.524594.

24. Ranstam J, Robertsson O. Statistical analysis of arthroplasty register data. Acta Orthop. 2010;**81**(1):10-4, doi: 10.3109/17453671003587168.

25. National Joint Registry. Knee revision procedure form MDS version 6. Secondary Knee revision procedure form MDS version 6 2016.

For peer review only

http://www.njrcentre.org.uk/njrcentre/Portals/0/Documents/England/Data%20collection%20forms/MDSv6.0_K2_V004.pdf.

26. Gundtoft PH, Overgaard S, Schonheyder HC, et al. The "true" incidence of surgically treated deep prosthetic joint infection after 32,896 primary total hip arthroplasties: a prospective cohort study. Acta Orthop. 2015;**86**(3):326-34, doi: 10.3109/17453674.2015.1011983.
27. Gundtoft PH, Pedersen AB, Schonheyder HC, et al. One-year incidence of prosthetic joint infection in total hip arthroplasty: a cohort study with linkage of the Danish Hip Arthroplasty Register and Danish Microbiology Databases. Osteoarthritis Cartilage 2016, doi: 10.1016/j.joca.2016.12.010.
28. Gundtoft PH, Pedersen AB, Schonheyder HC, et al. Validation of the diagnosis 'prosthetic joint infection' in the Danish Hip Arthroplasty Register. Bone Joint J 2016;**98-B**(3):320-5, doi: 10.1302/0301-620X.98B3.36705.
29. Lindgren JV, Gordon M, Wretenberg P, et al. Validation of reoperations due to infection in the Swedish Hip Arthroplasty Register. BMC Musculoskelet. Disord. 2014;**15**:384, doi: 10.1186/1471-2474-15-384.
30. Jamsen E, Varonen M, Huhtala H, et al. Incidence of prosthetic joint infections after primary knee arthroplasty. J. Arthroplasty 2010;**25**(1):87-92, doi: 10.1016/j.arth.2008.10.013.
31. Peersman G, Laskin R, Davis J, et al. Infection in total knee replacement: a retrospective review of 6489 total knee replacements. Clin. Orthop. Relat. Res. 2001(392):15-23.
32. Jamsen E, Huotari K, Huhtala H, et al. Low rate of infected knee replacements in a nationwide series--is it an underestimate? Acta Orthop. 2009;**80**(2):205-12, doi: 10.3109/17453670902947432.
33. Deirmengian C, Kardos K, Kilmartin P, et al. Combined measurement of synovial fluid alpha-Defensin and C-reactive protein levels: highly accurate for diagnosing periprosthetic joint infection. J. Bone Joint Surg. 2014;**96**(17):1439-45, doi: http://dx.doi.org/10.2106/JBJS.M.01316.
34. Paxton EW, Inacio MC, Khatod M, et al. Risk calculators predict failures of knee and hip arthroplasties: findings from a large health maintenance organization. Clin. Orthop. Relat. Res. 2015;**473**(12):3965-73, doi: 10.1007/s11999-015-4506-4.
35. Kaier K, Mutters NT, Frank U. Bed occupancy rates and hospital-acquired infections--should beds be kept empty? Clin. Microbiol. Infect. 2012;**18**(10):941-5, doi: 10.1111/j.1469-0691.2012.03956.x.

For peer review only

36. Kurtz SM, Lau E, Schmier J, et al. Infection burden for hip and knee arthroplasty in the United States. J. Arthroplasty 2008;**23**(7):984-91, doi: 10.1016/j.arth.2007.10.017.

37. Bohl DD, Samuel AM, Basques BA, et al. How much do adverse event rates differ between primary and revision total joint arthroplasty? J. Arthroplasty 2015, doi: 10.1016/j.arth.2015.09.033.

38. Pugely AJ, Martin CT, Gao Y, et al. The incidence of and risk factors for 30-day surgical site infections following primary and revision total joint arthroplasty. J. Arthroplasty 2015;**30**(9 Suppl):47-50, doi: 10.1016/j.arth.2015.01.063.

39. Rasouli MR, Restrepo C, Maltenfort MG, et al. Risk factors for surgical site infection following total joint arthroplasty. J. Bone Joint Surg. Am. 2014;**96**(18):e158, doi: 10.2106/jbjs.m.01363.

40. Tayton ER, Frampton C, Hooper GJ, et al. The impact of patient and surgical factors on the rate of infection after primary total knee arthroplasty : an analysis of 64 566 joints from the New Zealand Joint Registry. Bone Joint J 2016;**98-B**(3):334-40, doi: 10.1302/0301-620X.98B3.36775.

41. Nichols CI, Vose JG. Clinical Outcomes and Costs Within 90 Days of Primary or Revision Total Joint Arthroplasty. J. Arthroplasty 2016, doi: 10.1016/j.arth.2016.01.022.

42. Mahomed NN, Barrett J, Katz JN, et al. Epidemiology of total knee replacement in the United States Medicare population. J. Bone Joint Surg. Am. 2005;**87**(6):1222-8.

43. Hartley RC, Barton-Hanson NG, Finley R, et al. Early patient outcomes after primary and revision total knee arthroplasty. A prospective study. J. Bone Joint Surg. Br. 2002;**84**(7):994-9.

44. Lavernia C, Lee DJ, Hernandez VH. The increasing financial burden of knee revision surgery in the United States. Clin. Orthop. Relat. Res. 2006;**446**:221-6.

45. Kallala RF, Vanhegan IS, Ibrahim MS, et al. Financial analysis of revision knee surgery based on NHS tariffs and hospital costs: does it pay to provide a revision service? Bone Joint J 2015;**97-B**(2):197-201, doi: 10.1302/0301-620x.97b2.33707.

46. Wolf CF, Gu NY, Doctor JN, et al. Comparison of one and two-stage revision of total hip arthroplasty complicated by infection: a Markov expected-utility decision analysis. J. Bone Joint Surg. Am. 2011;**93-A**(7):631-9.

47. Nagra NS, Hamilton TW, Ganatra S, et al. One-stage versus two-stage exchange arthroplasty for infected total knee arthroplasty: a systematic review. Knee Surg. Sports Traumatol. Arthrosc. 2015.

48. Jamsen E, Stogiannidis I, Malmivaara A, et al. Outcome of prosthesis exchange for infected knee arthroplasty: the effect of treatment approach. Acta Orthop. 2009;**80**(1):67-77, doi: 10.1080/17453670902805064.

For peer review only

49. Patel A, Pavlou G, Mujica-Mota RE, et al. The epidemiology of revision total knee and hip arthroplasty in England and Wales: a comparative analysis with projections for the United States. A study using the National Joint Registry dataset. Bone Joint J 2015;**97-B**(8):1076-81, doi: 10.1302/0301-620X.97B8.35170.

For peer review only

LIST OF ABBREVIATIONS

PJI: Prosthetic joint infection;

NJR: National Joint Registry for England, Wales and Northern Ireland

BMI: Body Mass Index

ASA grade: American Society of Anesthesiologists grade

RR: Rate Ratio;

95% CI: 95% Confidence Interval

ADDITIONAL FILES

Web appendix Table 1: Characteristics of patients with a completed or partially-completed two-stage revision total knee replacement

FIGURE LEGENDS

Figure 1: Description of procedures recorded in the National Joint Registry.

Figure 2: Prevalence (95% CI) of revision for prosthetic joint infection within 2 years from the index primary surgery.

Figure 3. Prevalence (95% CI) of revision for prosthetic joint infection within 2 years from the index aseptic revision surgery.

Figure 4 : Cumulative Incidence Function of revision for prosthetic joint infection following index primary and aseptic revision knee replacement.

ADDITIONAL FIGURE LEGENDS

Web Appendix Fig 1. Prevalence (95% CI) of revision for prosthetic joint infection within 2 to 6 years

For peer review only

For peer review only - http://bmjopen.bmj.com/site/about/guidelines.xhtml

from the index primary surgery.

Web appendix Fig 2. Prevalence (95% CI) of revision for prosthetic joint infection within 2 to 6 years from the index aseptic revision surgery.

For peer review only

For peer review only - http://bmjopen.bmj.com/site/about/guidelines.xhtml

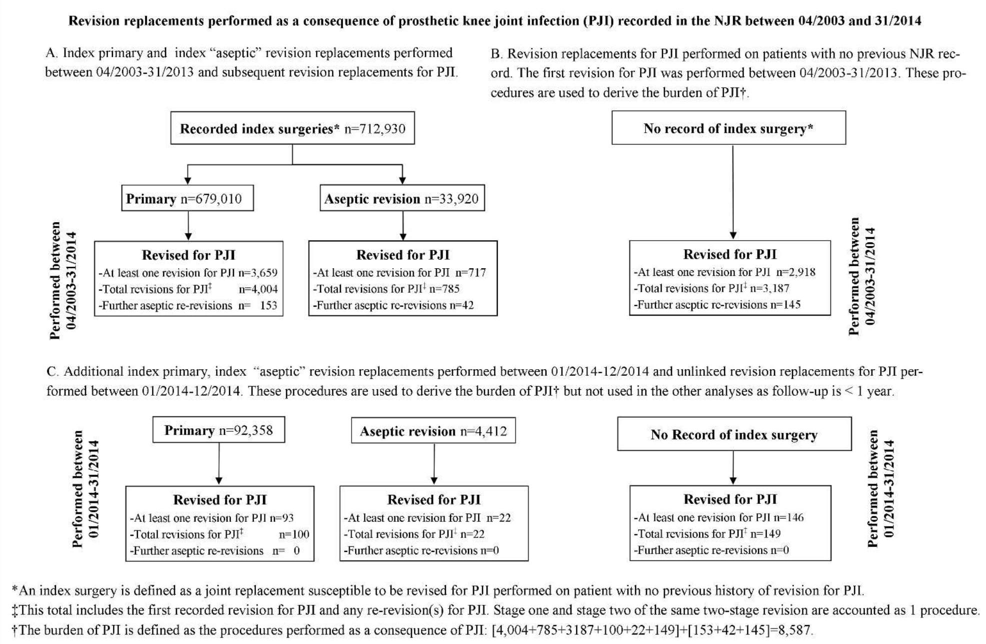

Figure 1

Figure 1

297x209mm (300 x 300 DPI)

iew only

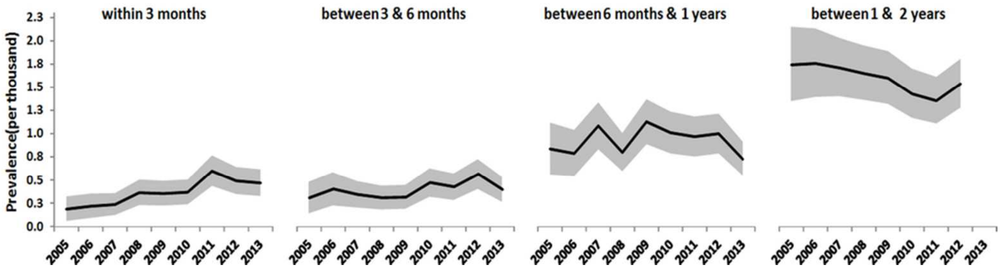

Figure 2
Figure 2
40x10mm (600 x 600 DPI)

For peer review only

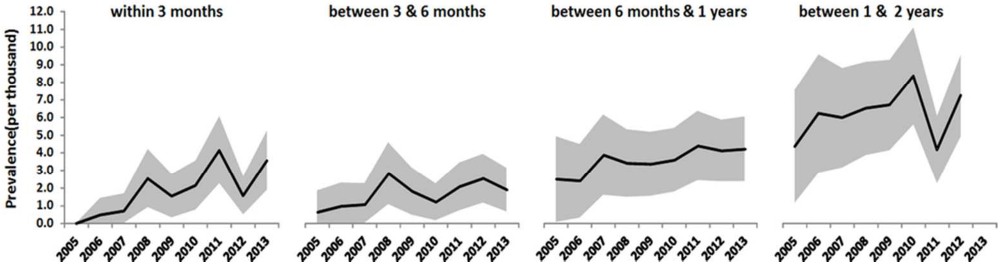

Figure 3
Figure 3
40x10mm (600 x 600 DPI)

For peer review only

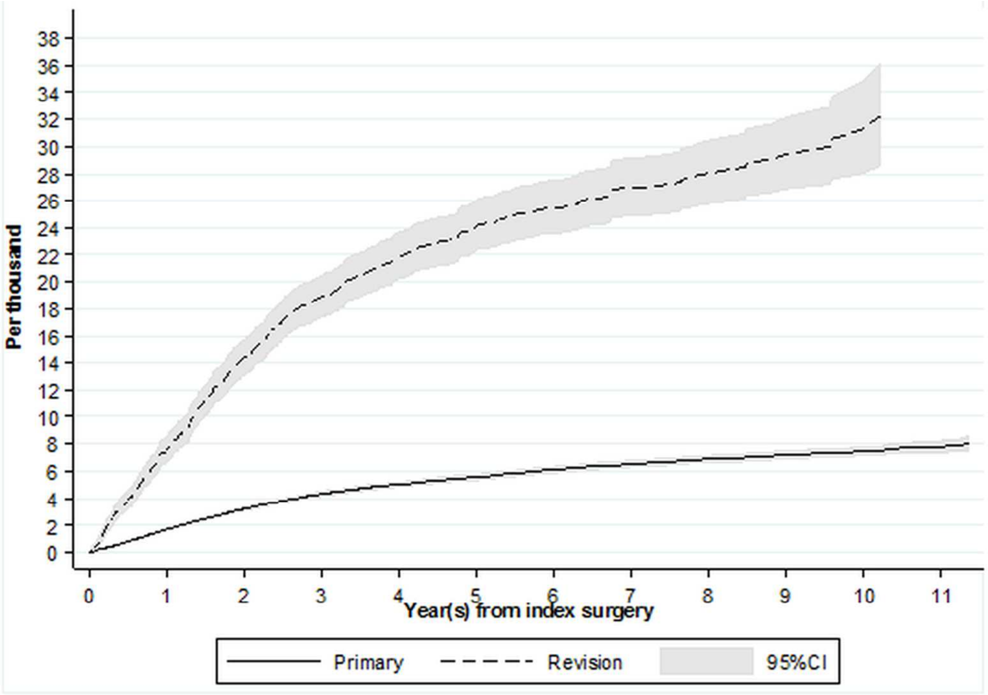

Figure 4
Figure 4
56x39mm (600 x 600 DPI)

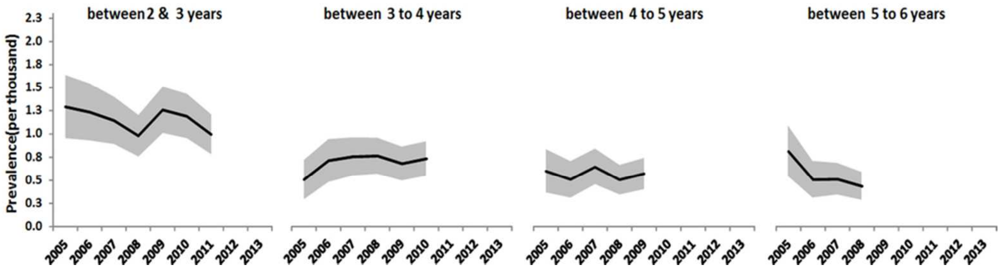

Web appendix Figure 1
Web appendix Figure1
40x10mm (600 x 600 DPI)

For peer review only

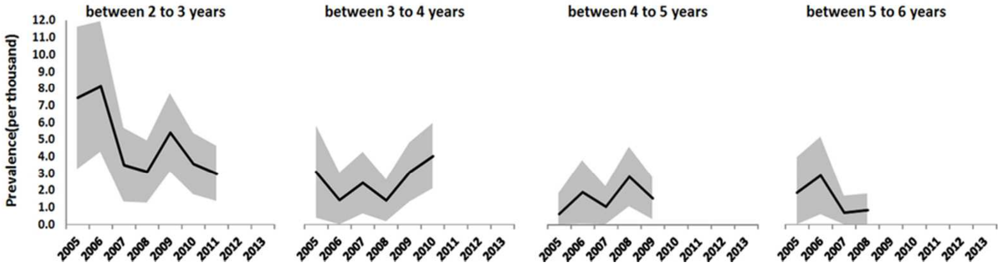

Web appendix Figure 2
Web appendix Figure 2
40x10mm (600 x 600 DPI)

For peer review only

Appendix Table 1: Characteristics of patients with a completed or partially-completed two-stage revision total knee replacement †

	Complete*	Only stage two**	Only stage one**
**Primary(n=2,~~833~~880)**	1,~~478~~512	792	~~563~~576
Age(mean, SD)	66(9)	67(9)	67(10)
Female(%)	44	41	43
BMI(mean, SD)	32(6)	32(6)	32(6)
missing BMI (n=)	~~308~~319	308	~~201~~204
ASA 1(%)	14	14	10
ASA 2(%)	68	67	64
ASA 3(%)	18	19	25
ASA 4-5(%)	0	0	1
**Revision(n=~~576~~589)**	~~294~~309	154	~~128~~126
Age(mean, SD)	66(10)	66(10)	~~69~~68(10)
Female(%)	40	41	33
BMI(mean, SD)	32(6)	31(6)	32(6)
missing BMI	~~59~~63	58	~~36~~32
ASA 1(%)	14	10	~~13~~14
ASA 2(%)	67	67	~~59~~58
ASA 3(%)	19	22	27
ASA 4-5(%)	0	1	1

†2,~~833~~ 880 two-stage revision total knee replacement procedures to manage an infection were recorded in the NJR following an index primary knee replacement and ~~576~~ 589 following an index aseptic revision surgery.

* Both stage one and stage two procedures of a two-stage revision total knee replacement reported in the NJR.

**Only stage one or stage two procedure of a two-stage revision total knee replacement reported in the NJR.

For peer review only

Appendix Table 1: Characteristics of patients with a completed or partially-completed two-stage revision total knee replacement †

	Complete*	Only stage two**	Only stage one**
**Primary(n=2,880)**	1,512	792	576
Age(mean, SD)	66(9)	67(9)	67(10)
Female(%)	44	41	43
BMI(mean, SD)	32(6)	32(6)	32(6)
missing BMI (n=)	319	308	204
ASA 1(%)	14	14	10
ASA 2(%)	68	67	64
ASA 3(%)	18	19	25
ASA 4-5(%)	0	0	1
**Revision(n=589)**	309	154	126
Age(mean, SD)	66(10)	66(10)	68(10)
Female(%)	40	41	33
BMI(mean, SD)	32(6)	31(6)	32(6)
missing BMI	63	58	32
ASA 1(%)	14	10	14
ASA 2(%)	67	67	58
ASA 3(%)	19	22	27
ASA 4-5(%)	0	1	1

†2,880 two-stage revision total knee replacement procedures to manage an infection were recorded in the NJR following an index primary knee replacement and 589 following an index aseptic revision surgery.

* Both stage one and stage two procedures of a two-stage revision total knee replacement reported in the NJR.

**Only stage one or stage two procedure of a two-stage revision total knee replacement reported in the NJR.

For peer review only

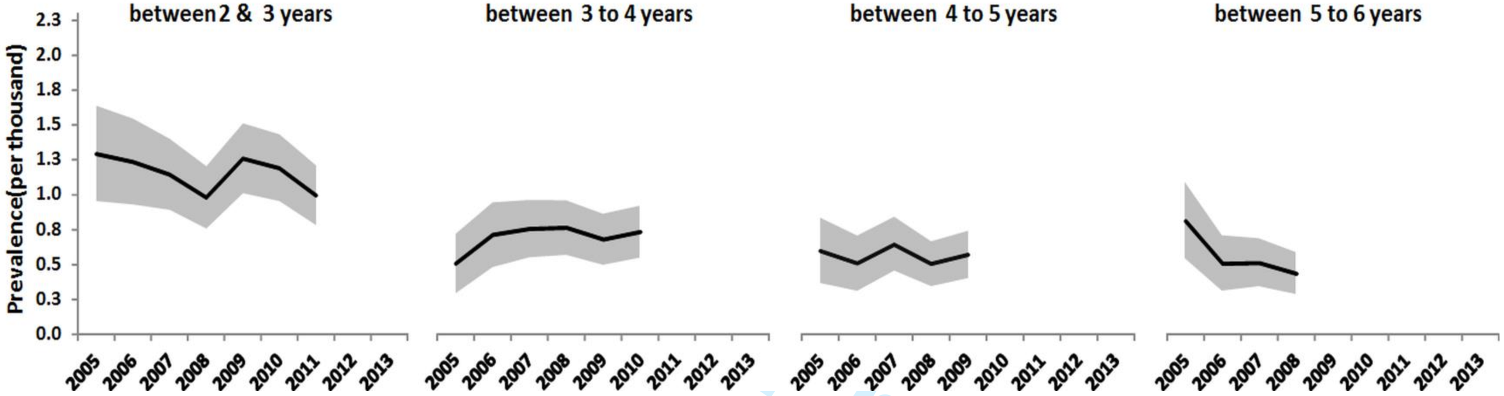

Web Appendix Fig 1. Prevalence (95% CI) of revision for prosthetic joint infection within 2 to 6 years from the index primary surgery.

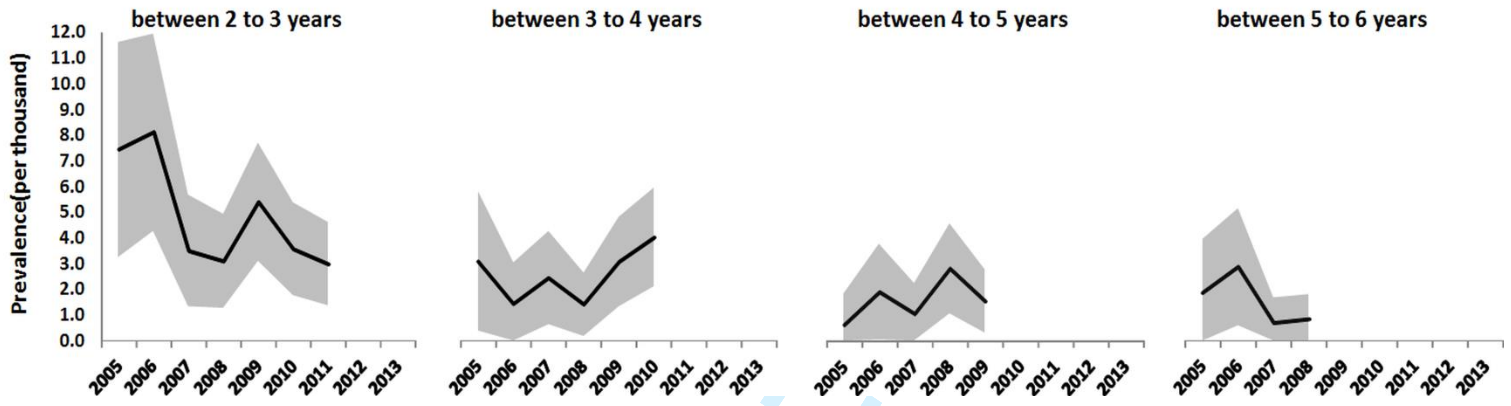

Web appendix Fig 2. Prevalence (95% CI) of revision for prosthetic joint infection within 2 to 6 years from the index aseptic revision surgery.

STROBE 2007 (v4) checklist of items to be included in reports of observational studies in epidemiology*
Checklist for cohort, case-control, and cross-sectional studies (combined)

Section/Topic	Item #	Recommendation	Checked
**Title and abstract**	1	(*a*) Indicate the study's design with a commonly used term in the title or the abstract	**Page 1**
		(*b*) Provide in the abstract an informative and balanced summary of what was done and what was found	**Pages 2-3**
**Introduction**			
Background/rationale	2	Explain the scientific background and rationale for the investigation being reported	**Pages 5-6**
Objectives	3	State specific objectives, including any pre-specified hypotheses	**Page 6**
**Methods**			
Study design	4	Present key elements of study design early in the paper	**Page 7**
Setting	5	Describe the setting, locations, and relevant dates, including periods of recruitment, exposure, follow-up, and data collection	**Pages 7-8+Figure 1**
Participants	6	Give the eligibility criteria, and the sources and methods of selection of participants. Describe methods of follow-up	**Page 7-8**
		For matched studies, give matching criteria and number of exposed and unexposed	na
Variables	7	Clearly define all outcomes, exposures, predictors, potential confounders, and effect modifiers. Give diagnostic criteria, if applicable	**Pages 7-8**
Data sources/ measurement	8*	For each variable of interest, give sources of data and details of methods of assessment (measurement). Describe comparability of assessment methods if there is more than one group	**Page 7-8**
Bias	9	Describe any efforts to address potential sources of bias	**Pages 8-9**
Study size	10	Explain how the study size was arrived at	**Page 7-8**
Quantitative variables	11	Explain how quantitative variables were handled in the analyses. If applicable, describe which groupings were chosen and why	**Pages 9-10**
Statistical methods	12	(*a*) Describe all statistical methods, including those used to control for confounding	**Pages 9-10**
		(*b*) Describe any methods used to examine subgroups and interactions	**Pages 9-10**
		(*c*) Explain how missing data were addressed	**Pages 8-9**
		(*d*) If applicable, explain how loss to follow-up was addressed	**Pages 8-9+Figure 1**
		(*e*) Describe any sensitivity analyses	na
**Results**			
Participants	13*	(a) Report numbers of individuals at each stage of study—eg numbers potentially eligible, examined for eligibility, confirmed eligible, included in the study, completing follow-up, and analysed	**Figure 1+Page 11**

For peer review only

		(b) Give reasons for non-participation at each stage	**Figure 1+Page 11**
		(c) Consider use of a flow diagram	**Figure 1+Page 11**
Descriptive data	14*	(a) Give characteristics of study participants (eg demographic, clinical, social) and information on exposures and potential confounders	**Page 11**
		(b) Indicate number of participants with missing data for each variable of interest	na
		(c)Summarise follow-up time (eg, average and total amount)	**Figures 2-4+Appendix figures 1-2+pages 10-13**
Outcome data	15*	Report numbers of outcome events or summary measures over time	**Tables 1-2+pages 11-14**
Main results	16	(*a*) Give unadjusted estimates and, if applicable, confounder-adjusted estimates and their precision (eg, 95% confidence interval). Make clear which confounders were adjusted for and why they were included	**Pages 11-14**
		(*b*) Report category boundaries when continuous variables were categorized	na
Other analyses	17	Report other analyses done—eg analyses of subgroups and interactions, and sensitivity analyses	**Pages 11-14**
**Discussion**			
Key results	18	Summarise key results with reference to study objectives	**Page 16**
Limitations	19	Discuss limitations of the study, taking into account sources of potential bias or imprecision. Discuss both direction and magnitude of any potential bias	**Pages 16-17**
Interpretation	20	Give a cautious overall interpretation of results considering objectives, limitations, multiplicity of analyses, results from similar studies, and other relevant evidence	**Pages 17-19**
Generalisability	21	Discuss the generalisability (external validity) of the study results	**Pages 17-18**
**Other information**			
Funding	22	Give the source of funding and the role of the funders for the present study and, if applicable, for the original study on which the present article is based	**Page 20**

*Give information separately for cases and controls in case-control studies and, if applicable, for exposed and unexposed groups in cohort and cross-sectional studies.

Note: An Explanation and Elaboration article discusses each checklist item and gives methodological background and published examples of transparent reporting. The STROBE checklist is best used in conjunction with this article (freely available on the Web sites of PLoS Medicine at http://www.plosmedicine.org/, Annals of Internal Medicine at http://www.annals.org/, and Epidemiology at http://www.epidem.com/). Information on the STROBE Initiative is available at www.strobe-statement.org.

For peer review only

BMJ Open

Description of the rates, trends and surgical burden associated with revision for prosthetic joint infection following primary and revision knee replacement in England and Wales. An analysis of the National Joint Registry for England, Wales, Northern Ireland and the Isle of Man.

Journal:	*BMJ Open*
Manuscript ID	bmjopen-2016-014056.R2
Article Type:	Research
Date Submitted by the Author:	10-Mar-2017
Complete List of Authors:	Lenguerrand, Erik; University of Bristol School of Clinical Science, Musculoskeletal Research Unit<br>Whitehouse, Michael; University of Bristol School of Clinical Science, Musculoskeletal Research Unit<br>Beswick, Andrew; University of Bristol School of Clinical Science, Musculoskeletal Research Unit<br>Toms, Andrew ; Royal Devon and Exeter NHS Foundation Trust, Princess Elizabeth Orthopaedic Centre, Exeter Knee Reconstruction Unit,<br>Porter, Martyn; Wrightington, Wigan and Leigh NHS Foundation Trust, Wrightington Hospital, Centre for Hip Surgery<br>Blom, Ashley; University of Bristol School of Clinical Science, Musculoskeletal Research Unit
**Primary Subject Heading**:	Surgery
Secondary Subject Heading:	Epidemiology
Keywords:	Knee < ORTHOPAEDIC & TRAUMA SURGERY, Primary knee replacement, Revision knee replacement, prosthetic joint infection, Registry

SCHOLARONE™
Manuscripts

Description of the rates, trends and surgical burden associated with revision for prosthetic joint infection following primary and revision knee replacement in England and Wales. An analysis of the National Joint Registry for England, Wales, Northern Ireland and the Isle of Man.

*Erik Lenguerrand[1], *Michael R Whitehouse[1], Andrew D Beswick[1], Andrew D Toms[2], Martyn L Porter[3], Ashley W Blom[3], on behalf of the National Joint Registry for England, Wales, Northern Ireland and the Isle of Man.

* Authors contributed equally to this work

1. University of Bristol, School of Clinical Sciences, Musculoskeletal Research Unit, Bristol, UK
2. Royal Devon and Exeter NHS Foundation Trust, Princess Elizabeth Orthopaedic Centre, Exeter Knee Reconstruction Unit, Exeter, UK
3. Wrightington, Wigan and Leigh NHS Foundation Trust , Wrightington Hospital, Centre for Hip Surgery, Lancashire, WN6 9EP

Correspondence to:
Name: Dr Erik Lenguerrand
Address: Musculoskeletal Research Unit, 1st Floor Learning & Research Building, School of Clinical Sciences, University of Bristol, Southmead Hospital, Bristol, BS10 5NB, UK
Email: erik.lenguerrand@bristol.ac.uk Tel: 0117 414 7865 Fax: 0117 414 7924

Corresponding author:
Name: Prof Ashley W Blom
Address: Musculoskeletal Research Unit, 1st Floor Learning & Research Building, School of Clinical Sciences, University of Bristol, Southmead Hospital, Bristol, BS10 5NB, UK
Email: ashley.blom@nbt.nhs.uk Tel: 0117 414 7865

Co-corresponding author:
Name: Mr Martyn L Porter
Address: Centre for Hip Surgery, Wrightington Hospital, Lancashire, WN6 9EP, UK
Email: martynporter@hipkneeclinic.com Tel: 01257 266 166

Word count: 4152

For peer review only

ABSTRACT

Objectives

To describe the prevalence rates of revision surgery for the treatment of prosthetic joint infection (PJI) for patients undergoing knee replacement, their time trends, the cumulative incidence function of revision for PJI and estimate the burden of PJI at health service level.

Design

We analysed revision knee replacements performed due to a diagnosis of PJI and the linked index procedures recorded in the National Joint Registry from 2003–2014 for England and Wales. The cohort analysed consisted of 679,010 index primary knee replacements, 33,920 index revision knee replacements and 8,247 revision total knee replacements performed due to a diagnosis of PJI. The prevalence rates, their time-trends investigated by time from index surgery to revision for PJI, cumulative incidence functions, and the burden of PJI (total procedures) were calculated. Overall linear trends were investigated with log-linear regression.

Results

The incidence of revision total knee replacement due to PJI at two years was 3.2/1,000 following primary and 14.4/1,000 following revision knee replacement respectively. The prevalence of revision due to PJI in the 3 months following primary knee replacement has risen by 2.5-fold (95%CI 1·2–5·3) from 2005–2013 and 7.5-fold (95%CI 1·0–56.1) following revision knee replacement. Over 1,000 procedures/year are performed as a consequence of knee PJI, an increase of 2.8 from 2005-2013. Overall, 75% of revisions were two-stage with an increase in use of single stage from 7.9% in 2005 to 18.8% in 2014.

Conclusions

For peer review only

Although the risk of revision due to PJI following knee replacement is low, it is rising, and coupled with the established and further predicted increased incidence of both primary and revision knee replacement, this represents an increasing and substantial treatment burden for orthopaedic service delivery in England and Wales. This has implications for future service design and the funding of individual and specialist centres.

Keywords: Orthopaedics, Primary knee replacement, Revision knee replacement, prosthetic joint infection, Registry

For peer review only

STRENGHT AND LIMITATIONS OF THIS STUDY

- This study provides updated and nationally representative descriptive evidence for England and Wales on revision for prosthetic joint infection following knee replacements.

- Over 600,000 index primary replacements, 30,000 index revision replacements and 8,000 revision total replacements performed due to a diagnosis of infection are included in the analyses.

- Contemporary evidence regarding changes over time in the risk of revision for prosthetic joint infection, its burden, and the types of revision surgery used for managing prosthetic joint infection is provided.

- The diagnosis of prosthetic joint infection recorded in the registry is not validated against a recognised international standard and may therefore be subject to over or under reporting.

- Not all recorded revisions for prosthetic joint infection had an index primary or aseptic revision total knee replacement recorded in the National Joint Registry.

For peer review only

BACKGROUND

Deep prosthetic joint infection (PJI) is a rare but serious complication of total knee replacement. Patients experience severe pain, functional difficulties, poor quality of life and, if untreated, loss of the affected limb or death. [1-3]

Estimates of the incidence of PJI after primary knee replacement range between 0.85% in Germany, 1.0% in the UK, 1.4% in Finland and 2.2% in the USA.[4-7] In a study from Finland, about 1.14% of patients had a PJI within 2 years of their primary knee replacement with a further 0.27% of patients presenting with PJI later than 2 years after primary surgery.[6] After aseptic revision and more complex knee replacement surgeries, infection rates may be considerably higher.[8] There is a paucity of evidences from UK centres and the available studies are old and based on single-centre data.[4 9]

Bacteria adhere as biofilm to implants and peri-prosthetic tissues making the treatment of PJI difficult.[10] Patients with PJI after knee replacement and their treating surgeons face complex and protracted treatment pathways. Initial treatment may involve surgical debridement, antibiotics and implant retention (DAIR), particularly in early postoperative infections. However about 45-52% of patients receiving DAIR may subsequently need revision of their implants.[11] Rates of revision of implants following treatment with a DAIR may be lower with strict selection criteria [12], but larger single centre cohort studies suggest the rate remains around 20% by two years [13].Delays to effective infection control inherent in this process may lead to a poor post replacement outcome.[14]

For patients with acute PJI diagnosed before biofilm maturation occurs, DAIR with modular exchange is a reasonable treatment option but for the majority of patients outside of this window of opportunity, contemporary management involves surgical revision with extensive debridement and antibiotic treatment with either immediate implant replacement (single-stage) or delayed implant replacement (two-stage).

The two-stage revision procedure was the first method reported for treatment of PJI of the knee that aimed to restore a functioning, painless knee.[15] The period between operations allows targeted antibiotic treatment and monitoring of clinical status and inflammatory markers. However, patients require two distinct planned major surgeries and a period of several months with limited knee function and protracted distress, concern and uncertainty.[16] An alternate one-stage strategy was developed by the Endo-Klinik and its use reported from 1976.[17] Key to the single-stage revision strategy is

For peer review only

extensive debridement, identification of the infecting organisms and appropriate tailored antibiotic treatment.[18]

Pooled evidence from case-series suggests similar rates of re-infection after each treatment.[19] The decision to treat with a single-stage or two-stage procedure may be guided by microbiological tests, patient and surgical factors but ultimately the choice of revision method is largely that of the treating surgeon. [2]

The National Joint Registry for England, Wales Northern Ireland and the Isle of Man (NJR) is the largest joint replacement database in the world and has collected data since April 2003.[20]

We analysed this dataset to assess the burden of revision surgery for knee PJI between April 2003 and December 2013. In this period, data was available from England and Wales. Our specific aims were to:

- Present the surgeon's perspective by describing the prevalence rates of revision surgery for the treatment of knee PJI following primary and revision surgery for aseptic indications, and their time trends broken down by time from index surgery to revision for infection.
- Present the patient's perspective by deriving cumulative incidence functions by type of index surgery.
- Estimate the burden of revision surgery for knee PJI at a health service level by accounting for all of the registered revisions and re-revision surgeries performed for knee PJI.

For peer review only

METHODS

Data source

In this observational study, we report analyses of data from the NJR.[20] The registry was established in 2003 and includes details of primary and revision knee replacements performed in England and Wales. Data entry for Northern Ireland and the Isle of Man did not commence until 2013 and 2015 respectively and data linkage for those periods is limited, therefore they are excluded from this analysis.

Index surgeries and revision surgeries for PJI

We grouped procedures as index knee replacements and revision surgeries for treatment of knee PJI. Index surgeries included all primary procedures and all revision procedures performed for an indication other than infection. The index revision procedures have been labelled "aseptic revision" procedures to indicate they were not performed due to PJI. All index surgeries performed between 1st April 2003 and 31st December 2013 were included (to allow a minimum 12-months follow-up).

Information on microbiology results are not recorded in the NJR and infected index procedures were identified using subsequent revision performed for an indication of PJI. The diagnosis and treatment strategy for PJI is at the discretion of the surgeon according to local protocols and available information at the time of surgery. Revision surgeries performed as a consequence of PJI between 1st April 2003 and 31st December 2014 were considered. Revisions not performed for PJI but that were performed on a knee previously operated on due to PJI were not considered as index procedures, but were used alongside the revision and re-revision procedures for PJI to define the "burden of PJI". Non-surgical management of PJI and surgical procedures where no revision or modular exchange of implants is performed are not recorded in the NJR.

Revision procedures are reported in the NJR as a single stage, a stage one of a two-stage revision, a stage two of a two-stage revision procedure, a conversion to arthrodesis, or an amputation. DAIR procedures with modular exchange are recorded in the NJR dataset as single stage revision procedures. To identify which procedures recorded as single stage revisions were DAIRs with modular exchange as opposed to complete single stage revisions where implants fixed to bone are also revised, the component level data was considered for the index and revision procedures. Implant component labels are compulsory for all NJR records, the minimum dataset forms also contain fields for

For peer review only - http://bmjopen.bmj.com/site/about/guidelines.xhtml

components removed but this information is discretionary and limited to the brand removed, therefore does not allow component tracing unless a linked index procedure exists. Procedures were therefore defined on the basis of the data provided for components implanted at revision surgery. Procedures recorded as single stage revisions where only modular components were added (termed "meniscal component" in the minimum data set) were defined as DAIRs with modular exchange. Those where implants fixed to bone were implanted ("femoral component", "tibial tray" +/- "patella") were defined as single stage revisions. Debridements where the surgeon either elects to not exchange modular components when they are present or where modular exchange is not possible (e.g. with monoblock polyethylene tibial components) are not captured in the NJR.

Missing procedures

There were 2,880 two-stage revision procedures recorded where the index surgery was a primary knee replacement, in 792 (27%) of these, only a stage two of a two-stage procedure was recorded in the NJR. This was also observed for 154 (26%) of the 589 two-stage revision procedures following an index aseptic revision procedure. Patients with incompletely registered two-stage procedures did not differ from those with complete information in terms of gender, BMI, and ASA grade (see appendix Table 1). For index primary knee replacement, patients with incomplete information were on average one year older than those with complete information (p=0.05). For aseptic revision surgeries, no age difference was observed between the two groups.

For incomplete procedures, the date of the stage one of a two-stage revision procedure was estimated. Firstly we derived the relative weight of time elapsed between the index and stage one of a two-stage procedure by year and type of index surgery using patients with complete information: 100x(length of time$_{\text{index surgery- 1st stage}}$/length of time $_{\text{index surgery- 2nd stage}}$). We applied these weights to the length of time observed between the index and stage two of a two-stage procedure for those with incomplete information to obtain an estimate for the time between the index surgery and stage one of the revision surgery.

Statistical analyses

Analyses were performed with Stata SE 13.1 (StataCorp LP, TX, USA).

For peer review only - http://bmjopen.bmj.com/site/about/guidelines.xhtml

Surgeon perspective

Prevalence rates of index surgeries performed between 2003–2013 and subsequently revised for infection between 2003–2014 were derived by year and type of index surgery. This provides a "surgeon" perspective of revision for PJI by describing the proportion of knee replacements which required revision surgery for the management of infection. The prevalence rates were plotted by time from index surgery to revision for infection (≤3 months, 3–6 months, 6 months–1 year, 1–2 years, 2–3 years, 3–4 years, and 5–6 years). For two-stage surgeries, the date of stage one of a two-stage revision was selected to indicate the date of revision for PJI. Log-linear regression, using the year of the index knee replacement as a continuous independent factor, was used to investigate overall linear trends between 2005 and 2013. This period was selected as over 85% (proportion of procedure records submitted to the NJR compared with the levy returns for the number of implants sold) of knee replacements performed in 2005 and over 99% of those performed from 2007 onwards had been recorded in the NJR; prior to 2005, the data capture of the NJR was <75%.[21] When evidence of a time-trend was identified (year of surgery, $p \leq 0.05$), the year of surgery was re-considered as a categorical variable using 2005 as the reference period. Estimated relative risk and related 95% confidence intervals (95%CI) quantified the relative increase in prevalence rates between the period of interest and 2005.

Patient perspective

To move from a surgical perspective to a patient perspective, cumulative incidence functions [22-24] were derived by type of index surgery (primary or aseptic revision). These provide the probability of being revised as a consequence of PJI within a specific time period following the index surgery while accounting for the time patients were at risk of being revised for a PJI and the competing risks of death and revision for an aseptic indication.

For peer review only

Healthcare service perspective

The overall “burden of revision surgery for knee PJI” was analysed using all revision procedures performed between 2003 and 2014 for an indication of PJI as well as any subsequent re-revision procedures for PJI or another indication.

Typically, knee replacement survivorship is 10 years or more following surgery,[20] re-revision procedures performed for another indication than PJI during the observation period are therefore considered as a consequence of the earlier management of PJI.

Revision procedures for an indication of PJI with no index procedure recorded in the NJR (n=3,064, Figure 1) and their re-revision procedures were also accounted. Contrary to the previous analyses in which index surgeries performed in 2014 and their revisions procedures were not considered to allow for a minimum of one-year post-operative follow-up, revisions for PJI relating to index knee replacements performed in 2014 (n=122) were included in this analysis.

The overall burden was reported by the year and type of revision surgery. Stage one of a two-stage revision and stage two of a two-stage revision were considered as one procedure to avoid double counting. The same strategy was also used to account for multiple stage ones of a two-stage revision procedure.

For peer review only

RESULTS

Surgeon perspective

There were 679,010 primary and 33,920 aseptic revision knee replacements recorded in the NJR for England and Wales between April 2003 and December 2013 (Table 1).

A total of 3,659 patients required at least one revision surgery due to PJI (4,004 procedures) following a primary knee replacement (Figure 1.A). A further 717 patients required a subsequent revision for PJI (785 procedures) following an aseptic revision knee replacement (Figure 1.B). For 2,918 patients who underwent revision for PJI (3,187 procedures), no index surgery was recorded(Figure 1.C).

<Figure 1 here>

Figure 1. Description of procedures recorded in the National Joint Registry.

Of the primary knee replacements recorded in the NJR, 5.39/1,000 (95%CI 5.21-5.56) were subsequently revised due to PJI (Table 1). Of the aseptic revision knee replacements, 21.14/1,000(95%CI 19.61-22.67) were subsequently revised due to PJI.

For peer review only

Table 1. Primary and aseptic revision knee replacement procedures revised for a prosthetic knee joint infection (PJI).

Year of index procedure	Index primary replacement			Index aseptic revision replacement		
	Total N=	Revised for PJI n=	Prevalence rate Per 1,000, 95%CI	Total N=	Revised for PJI n=	Prevalence rate Per 1,000, 95%CI
**Total**	**679,010**	**3,659**	**5.39(5.21, 5.56)**	**33,920**	**717**	**21.14(19.61, 22.67)**
2003	13,547	112	8.27(6.74, 9.79)	534	24	44.94(27.37, 62.52)
2004	27,706	184	6.64(5.68, 7.60)	1,001	24	23.98(14.50, 33.45)
2005	41,848	314	7.50(6.68, 8.33)	1,612	41	25.43(17.75, 33.12)
2006	49,482	346	6.99(6.26, 7.73)	2,094	55	26.27(19.42, 33.12)
2007	66,618	471	7.07(6.43, 7.71)	2,847	60	21.07(15.80, 26.35)
2008	73,859	443	6.00(5.44, 6.55)	3,538	87	24.59(19.49, 29.69)
2009	75,612	465	6.15(5.59, 6.71)	3,884	91	23.43(18.67, 28.19)
2010	78,276	423	5.40(4.89, 5.92)	4,196	102	24.31(19.65, 28.97)
2011	81,795	378	4.62(4.16, 5.09)	4,336	81	18.68(14.65, 22.71)
2012	85,161	341	4.00(3.58, 4.43)	5,115	93	18.18(14.52, 21.84)
2013	85,106	182	2.14(1.83, 2.45)	4,763	59	12.39(9.25, 15.53)

For peer review only

For peer review only - http://bmjopen.bmj.com/site/about/guidelines.xhtml

Figure 2 shows trends in revision for PJI in the 2 years after primary knee replacement. Revision rates within 3 months of the index procedure increased over time, with the prevalence rate in 2013 over twice that in 2005, RR 2.46 (95%CI 1.15-5.25; time trend p<0.0001). No time-trends for revision rates between 2005 and 2013 were apparent in the periods 3–6 months, 6 months–1 year and 1–2 years. Other than the prevalence rate of revision for PJI between 5–6 years which decreased over time, RR 0·53 (95%CI 0.33-0.86; time trend p=0.018), there was no evidence of time-trends for the rates of late revision for PJI (Web appendix Figure1).

<Figure 2 here>

Figure 2. Prevalence (95%CI) of revision for prosthetic joint infection within 2 years of the index primary knee replacement.

Trends in subsequent revision of aseptic revision knee replacements for PJI are shown in Figure 3 and Web appendix Figure 2. The prevalence of aseptic revisions revised for PJI within 3 months of the procedure increased over time, RR 7.47 (95%CI 1.00-56.12; time trend p=0.001 for 2013 compared to 2006). No important differences were noted in the prevalence rates of revision for PJI performed at any time beyond 3 months from the index revision surgery (all time trend p-values >0.1) with the exception of revision for infection between 2–3 years for which the rates decreased over time, RR 0.40 (95%CI 0.18-0.88; time trend p=0.008 for 2011 compared to 2005).

<Figure 3 here>

Figure 3. Prevalence (95%CI) of revision for prosthetic joint infection within 2 years of the index aseptic revision knee replacement.

Patient perspective

Figure 4 shows the incidence over time of revision for PJI in patients with an index primary or aseptic revision while accounting for the risk of death and revision for any indication other than PJI. The probability of revision for PJI at one year following a primary knee replacement 1.7/1,000 (95%CI 1.6-1.8) compared to 7.6/1,000 (95%CI 6.8-8.6) following an aseptic revision knee replacement. At two years the probability was 3.2/1,000 (95%CI 3.1-3.4) and 14.4/1,000(95%CI 13.1-15.7) respectively, and at five years it was 5.6/1,000 (95%CI 5.4-5.8) and 24.1/1,000 (95%CI 22.3-26.1) respectively. The

For peer review only

For peer review only - http://bmjopen.bmj.com/site/about/guidelines.xhtml

probability of revision for infection within the first ten years following primary knee replacement was 7.5/1,000 (95%CI 7.2-7.8) and 31.3/1,000 (95%CI 28.1-24.9) following an aseptic revision.

<Figure 4 here>

Figure 4. Cumulative incidence function of revision for prosthetic joint infection following index primary and aseptic revision knee replacement.

Healthcare service perspective

Table 2 summarises the revision surgeries for the management of PJI and subsequent re-revisions (including repeated procedures to manage PJI and other revision procedures, see Figure 1 (A-C)) performed after primary and aseptic revision knee replacement by the year the (re-)revision was performed.

The absolute number of procedures performed as a consequence of PJI in England and Wales has increased from 378 in 2005 to 1,048 in 2014, a relative increase of 2.8 fold. This is higher than the 2.1 fold increase in primary knee replacements over the same period, but similar to the 2.9 increase in aseptic revision surgeries between 2005 and 2013 (table 1).

Overall, 75% of revisions were conducted with a two-stage procedure but the use of single-stage revision for PJI has increased from 7.9% in 2005 to 18.8% in 2014. The median interval between stages in a two-stage revision following a primary index surgery was 99 days (25th-75th percentiles: 68, 156). A 101 day (67, 147) median interval was observed for two-stage revision performed to manage PJI following an index aseptic revision surgery. The DAIR procedures represented around 5% of the total reported procedures and 9% of those reported in 2014. Other types of revision surgeries were rare and consisted mainly of arthrodesis (110 out of 134).

For peer review only

Table 2. Revision procedures performed as a consequence of prosthetic joint infection (PJI) by type and year of procedure *

Year of revision procedure	Total N=	DAIR n=, (%)	Single stage n=, (%)	Two-stage n=, (%)	Other† n=, (%)
**Total**	**8,587**	408(4.8)	1,589(18.5)	**6,456**(75.2)	**134**(1.6)
2003	126	3(2.4)	116(92.1)	7(05.6)	0(0.0)
2004	236	3(1.3)	59(25.0)	171(72.5)	3(1.3)
2005	378	8(2.1)	30(07.9)	336(88.9)	4(1.1)
2006	485	11(2.3)	49(10.1)	419(86.4)	6(1.2)
2007	663	19(2.9)	70(10.6)	560(84.5)	14(2.1)
2008	783	29(3.7)	124(15.8)	615(78.5)	15(1.9)
2009	852	32(3.8)	145(17.0)	662(77.7)	13(1.5)
2010	931	38(4.1)	178(19.1)	707(75.9)	8(0.9)
2011	1,004	51(5.1)	187(18.6)	748(74.5)	18(1.8)
2012	1,035	58(5.6)	200(19.3)	754(72.9)	23(2.2)
2013	1,046	61(5.8)	234(22.4)	732(70.0)	19(1.8)
2014	1,048	95(9.1)	197(18.8)	745(71.1)	11(1.1)

*This table reports the revision procedures performed after any index surgery revised as a consequence of PJI including subsequent re-revision procedures whether performed to manage an infection or not. It also includes the revision procedures performed between 2003 and 2014 on 3,064 patients with prosthetic joint infection but with no index procedure documented in the NJR.
† Conversion to arthrodesis or amputation

DISCUSSION

This study is the largest to date investigating the treatment of PJI after knee replacement. It includes 712,930 primary and aseptic revision index procedures and 8,247 revision total knee replacements performed due to a diagnosis of PJI. We have shown that the prevalence and incidence rates of revision for PJI are higher following aseptic revision knee replacement than for primary knee replacement and that the prevalence of revision for PJI within 3 months of surgery has risen over time for primary and aseptic revision procedures. The total burden of treating PJI of the knee has risen substantially, with over 1,000 procedures performed per year from 2011 onwards, which mirrors the greater rise in aseptic revision surgery compared to primary knee replacements.[20] From 2005, we have identified an increased use of single stage as compared to two-stage revision for PJI of the knee which appears to have plateaued at approximately 20%.

For peer review only

Strengths and weaknesses

The NJR is an established, large, prospective, observational arthroplasty register with comprehensive coverage of procedures undertaken, which is reassuring in terms of the generalisability of the data generated by the study. The data has some limitations. The recording of PJI as an indication for surgery is according to the opinion of the treating surgeon at the time of surgery and, as such, the diagnosis has not necessarily been referenced against a gold standard set of criteria and hence may be subject to misdiagnosis. Whilst the NJR now represents a mature dataset with long term follow up, there are still a substantial number of procedures undergoing revision knee replacement where there is no record of a linked index primary or revision knee replacement or where there are an incomplete set of procedures recorded for two stage revision surgery. This may be a reflection of the high survivorship of knee replacement for 10 years or more following surgery,[20] the fact that some PJI is acquired late and that not every case performed since 2003 in England and Wales has been recorded in the NJR. Given there was no difference between patients with complete two-stage episodes recorded and those with partial episodes recorded in terms of age, gender, BMI and ASA grade, we have assumed that the time interval of the former is generalisable to the latter, which may not be the case. The NJR does not capture information on microbiology results, non-surgical treatment of PJI and revision for PJI with implant retention but no modular exchange. Currently in the NJR, the only option to record DAIR procedures where modular exchange is performed is to record them as single stage revision surgeries.[25] This complicates their differentiation from single stage surgeries where the femoral and tibial components are revised. This represents a potential weakness of the data collection form as this may be subject to different interpretation by surgeons, despite the fact that recording of procedures in which any component is removed or inserted is mandatory. We have used component level data for individual cases to identify DAIR procedures with modular exchange. A few single-stage revisions for PJI unlinked to an index procedure were considered as DAIRs in view of the components implanted (n=66). The capture of revision surgeries, in particular those performed for PJI is also not perfect in arthroplasty registries [26-29] and it is unclear how complete the capture of these procedures in the NJR is.

The annual burden of PJI with over 1,000 procedures in recent years, while already high and expensive, is a conservative estimation and the cost of PJI considerable for the NHS.

For peer review only - http://bmjopen.bmj.com/site/about/guidelines.xhtml

Comparison with other studies

Revision for infection is a rare complication of knee replacement. Our findings in this respect are consistent with previous studies from England and Wales [4 9] although the reported prevalence rates were higher in previous studies (around 1% at 5 years following primary replacement compared to 0.5% in our study). Our study was not contemporaneous with those studies (index surgeries performed between 1987-2001 [9] or 1993-1996 [4]) and the considered sample size in them was smaller (n=4,788 and n=931). Evidence from other countries shows similar prevalence rates of revision for infection (rates ranging from 0.4% to 2.2%).[5-7 30 31] Studies which report incidence rates typically include any type of operations for the management of infection (not just those where implants are removed or changed) and this may be why they show higher rates (0.9% to 1.4% between 1 and 5 years postoperatively).[6 32]

We have demonstrated a substantial time trend of an increased risk of revision for PJI within the first 3 months of an index primary or aseptic revision knee replacement being performed. This phenomenon is likely to be multifactorial. Factors that could lead to an increased diagnosis of PJI in this period and hence increased risk of revision include the increased accuracy of tests available to clinicians for the diagnosis of PJI,[33] coupled with more rapid diagnosis and/or treatment in specialist centres, the increased risk factors for PJI amongst the population undergoing knee replacement population (such as elevated body mass index [34]) and increased bed occupancy within the healthcare setting in which these procedures were performed.[35] There may be a trend towards the use of revision surgery to manage PJI rather than suppressive treatment with antibiotics but we cannot comment on this as non-surgical management of PJI is beyond the scope of this study. There have been similar findings in other countries that could not be accounted for by risk factors recorded in those registries, suggesting this trend could reflect an actual general increase in the risk of PJI. [36] It is interesting to note from our results that there has not been a relative increase in the use of DAIRs with modular exchanges in the NJR to explain this phenomenon.

We have shown a greater incidence of revision for PJI following aseptic revision knee replacement (3.1% at 10 years) compared to primary knee replacement (0.8% at 10 years) consistent with the findings of previous cohort studies.[4 31 32 37-39] Previous surgery is a known risk factor for PJI, [40]

For peer review only - http://bmjopen.bmj.com/site/about/guidelines.xhtml

this may because of further bacterial contamination or increased length of surgery. Revision knee replacement tends to be performed in a population with increased host [41] and procedure risk factors for infection or further revision [42] and involve the introduction of a greater volume of prosthetic material and additional adjuncts such as bone graft that may present a favourable environment for bacterial colonisation and subsequent PJI. [43]

Implications for clinicians and policymakers

The current rate of surgical revision of PJI of the knee stands at approximately 1,000 cases per year across the NJR. This represents a significant healthcare burden which has more than doubled over the last decade, exceeding the rate of increase in primary knee replacements. The cost of revision knee replacement due to PJI is more than three times that of primary knee replacement and over twice that of aseptic revision.[44] In the National Health Service, the costs associated with revision knee replacement due to PJI have been shown to be in excess of £30,000 per case, greater than other indications for revision,[45] even before accounting for costs associated with litigation. Single stage revision offers an advantage over the two-stage approach in terms of both patient and surgeon derived utility values in both the short and long term in the hip [46] and may be associated with superior functional outcomes in the knee [47] suggesting this increased burden could be ameliorated by the increased use of a single stage revision strategy.

Unanswered questions and future research

The observed trend of an increase in the risk of infection in the first 3 months following primary and aseptic revision total knee replacement is not currently well understood. Identification of the risk factors associated with this phenomenon and modifiable risk factors for PJI may help to reduce this risk. Although the utilisation of single stage revision knee replacement for the treatment of PJI has increased to approximately 20%, given the current evidence base shows equivalent reinfection rates following single stage compared to two-stage revision surgery for PJI,[19 48] it might be possible that the proportion of patients treated with single-stage surgery taking account of patient, surgeon, and operative factors could be further increased. Given the increasing burden of treating PJI, this may help ameliorate the increase in resources that will otherwise be required to treat this condition [49]. In the

For peer review only

absence of data from randomised controlled trials or other direct comparisons, we plan further analyses of the NJR data which will compare outcomes after one- and two-stage methods with robust adjustment for key patient and surgical factors. Such evidence will support the decision making process in the planning and treatment of patients with PJI after knee replacement.

For peer review only

DECLARATIONS

Acknowledgments

We thank the patients and staff of all the hospitals who have contributed data to the National Joint Registry. We are grateful to the Healthcare Quality Improvement Partnership, the National Joint Registry Steering Committee, and staff at the National Joint Registry for facilitating this work.

Competing interest

MP is the Medical Director of the National Joint Registry and also acts as Chair of the Programme Steering Committee for the National Institute for Health Research (NIHR) INFORM program (PGfAR program: RP-PG-1210-12005). The other authors have no conflicts of interest.

Funding statement

This article presents independent research funded by the National Institute for Health Research (NIHR) under its Programme Grants for Applied Research program (RP-PG-1210-12005). The funders had no role in study design, data collection and analysis, decision to publish, or preparation of the manuscript.

Authors' contributions

ADB, AWB, EL and MRW designed the study. ADB, AWB, EL, and MRW reviewed the published work. The data were extracted by Northgate (Hemel Hempstead, UK). EL has full access to all the data and AWB is the guarantor. EL conducted the statistical analyses. ADB, ADT, AWB, EL, MLP and MRW interpreted the data. ADB, AWB, EL and MRW drafted the article. ADB, ADT, AWB, EL, MLP and MRW reviewed the article and gave their final approval. ADB, ADT, AWB and EL obtained the funding for this research.

For peer review only

Disclaimer

The views expressed represent those of the authors and do not necessarily reflect those of the National Joint Registry Steering Committee or Healthcare Quality Improvement Partnership, who do not vouch for how the information is presented.

The views expressed in this article are those of the authors and not necessarily those of the NHS, the NIHR, or the Department of Health.

Ethic approval and consent to participate

Patient consent was obtained for data collection by the National Joint Registry. According to the specifications of the NHS Health Research Authority, separate informed consent and ethical approval were not required for the present study.

Data Sharing StatementAccess to the data analysed in this study required permission from the National Joint Registry for England, Wales and Northern Ireland Research Sub-committee. http://www.njrcentre.org.uk/njrcentre/Research/Researchrequests/tabid/305/Default.aspx contains information on research data access request to the National Joint Registry.

For peer review only

References

1. Cahill JL, Shadbolt B, Scarvell JM, et al. Quality of life after infection in total joint replacement. J Orthop Surg 2008;**16**(1):58-65.
2. Gehrke T, Alijanipour P, Parvizi J. The management of an infected total knee arthroplasty. Bone Joint J 2015;**97-B**(10 Suppl A):20-9, doi: 10.1302/0301-620X.97B10.36475.
3. Zmistowski B, Karam JA, Durinka JB, et al. Periprosthetic joint infection increases the risk of one-year mortality. J. Bone Joint Surg. Am. 2013;**95**(24):2177-84, doi: http://dx.doi.org/10.2106/JBJS.L.00789.
4. Blom AW, Brown J, Taylor AH, et al. Infection after total knee arthroplasty. J. Bone Joint Surg. Br. 2004;**86-B**(5):688-91.
5. Meyer E, Weitzel-Kage D, Sohr D, et al. Impact of department volume on surgical site infections following arthroscopy, knee replacement or hip replacement. BMJ Qual Saf 2011;**20**(12):1069-74, doi: 10.1136/bmjqs.2011.051250.
6. Huotari K, Peltola M, Jamsen E. The incidence of late prosthetic joint infections: a registry-based study of 112,708 primary hip and knee replacements. Acta Orthop. 2015;**86**(3):321-5, doi: 10.3109/17453674.2015.1035173.
7. Kurtz SM, Lau E, Watson H, et al. Economic burden of periprosthetic joint infection in the United States. J. Arthroplasty 2012;**27**(8 Suppl):61-5, doi: 10.1016/j.arth.2012.02.022.
8. Mortazavi SM, Schwartzenberger J, Austin MS, et al. Revision total knee arthroplasty infection: incidence and predictors. Clin. Orthop. Relat. Res. 2010;**468**(8):2052-9, doi: 10.1007/s11999-010-1308-6.
9. Phillips JE, Crane TP, Noy M, et al. The incidence of deep prosthetic infections in a specialist orthopaedic hospital: a 15-year prospective survey. J. Bone Joint Surg. Br. 2006;**88**(7):943-8.
10. Arnold WV, Shirtliff ME, Stoodley P. Bacterial biofilms and periprosthetic infections. J. Bone Joint Surg. Am. 2013;**95**(24):2223-9.
11. Romano CL, Manzi G, Logoluso N, et al. Value of debridement and irrigation for the treatment of peri-prosthetic infections. A systematic review. Hip Int. 2012;**22 Suppl 8**:S19-24, doi: http://dx.doi.org/10.5301/HIP.2012.9566.
12. Laffer RR, Graber P, Ochsner PE, et al. Outcome of prosthetic knee-associated infection: evaluation of 40 consecutive episodes at a single centre. Clin. Microbiol. Infect. 2006;**12**(5):433-9.

For peer review only

13. Byren I, Bejon P, Atkins BL, et al. One hundred and twelve infected arthroplasties treated with 'DAIR' (debridement, antibiotics and implant retention): antibiotic duration and outcome. J. Antimicrob. Chemother. 2009;**63**(6):1264-71, doi: 10.1093/jac/dkp107.

14. Sherrell JC, Fehring TK, Odum S, et al. Fate of two-stage reimplantation after failed irrigation and debridement for periprosthetic knee infection. Clin. Orthop. Relat. Res. 2011;**469**(1):18-25.

15. Insall JN, Thompson FM, Brause BD. Two-stage reimplantation for the salvage of infected total knee arthroplasty. J. Bone Joint Surg. Am. 1983;**65**(8):1087-98.

16. Moore AJ, Blom AW, Whitehouse MR, et al. Deep prosthetic joint infection: a qualitative study of the impact on patients and their experiences of revision surgery. BMJ Open 2015;**5**(12):e009495, doi: 10.1136/bmjopen-2015-009495.

17. von Foerster G, Kluber D, Kabler U. Mid- to long-term results after treatment of 118 cases of periprosthetic infections after knee joint replacement using one-stage exchange surgery. Orthopade 1991;**20**(3):244-52.

18. Tibrewal S, Malagelada F, Jeyaseelan L, et al. Single-stage revision for the infected total knee replacement: results from a single centre. Bone Joint J 2014;**96-B**(6):759-64, doi: 10.1302/0301-620X.96B6.33086.

19. Kunutsor SK, Whitehouse MR, Lenguerrand E, et al. Re-Infection Outcomes Following One- And Two-Stage Surgical Revision of Infected Knee Prosthesis: A Systematic Review and Meta-Analysis. PLoS One 2016;**11**(3):e0151537, doi: 10.1371/journal.pone.0151537.

20. *National Joint Registry for England, Wales and Northern Ireland: 12th Annual Report*. Hemel Hempstead: NJR Centre 2015.

21. *National Joint Registry for England and Wales: 9th Annual Report.* Hemel Hempstead: NJR Centre, 2012.

22. Coviello V, M. B. Cumulative incidence estimation in the presence of competing risks. Stata Journal 2004(4):103–12.

23. Gillam MH, Ryan P, Graves SE, et al. Competing risks survival analysis applied to data from the Australian Orthopaedic Association National Joint Replacement Registry. Acta Orthop. 2010;**81**(5):548-55, doi: 10.3109/17453674.2010.524594.

24. Ranstam J, Robertsson O. Statistical analysis of arthroplasty register data. Acta Orthop. 2010;**81**(1):10-4, doi: 10.3109/17453671003587168.

25. National Joint Registry. Knee revision procedure form MDS version 6. Secondary Knee revision procedure form MDS version 6 2016.

For peer review only

http://www.njrcentre.org.uk/njrcentre/Portals/0/Documents/England/Data%20collection%20forms/MDSv6.0_K2_V004.pdf.

26. Gundtoft PH, Overgaard S, Schonheyder HC, et al. The "true" incidence of surgically treated deep prosthetic joint infection after 32,896 primary total hip arthroplasties: a prospective cohort study. Acta Orthop. 2015;**86**(3):326-34, doi: 10.3109/17453674.2015.1011983.

27. Gundtoft PH, Pedersen AB, Schonheyder HC, et al. One-year incidence of prosthetic joint infection in total hip arthroplasty: a cohort study with linkage of the Danish Hip Arthroplasty Register and Danish Microbiology Databases. Osteoarthritis Cartilage 2016, doi: 10.1016/j.joca.2016.12.010.

28. Gundtoft PH, Pedersen AB, Schonheyder HC, et al. Validation of the diagnosis 'prosthetic joint infection' in the Danish Hip Arthroplasty Register. Bone Joint J 2016;**98-B**(3):320-5, doi: 10.1302/0301-620X.98B3.36705.

29. Lindgren JV, Gordon M, Wretenberg P, et al. Validation of reoperations due to infection in the Swedish Hip Arthroplasty Register. BMC Musculoskelet. Disord. 2014;**15**:384, doi: 10.1186/1471-2474-15-384.

30. Jamsen E, Varonen M, Huhtala H, et al. Incidence of prosthetic joint infections after primary knee arthroplasty. J. Arthroplasty 2010;**25**(1):87-92, doi: 10.1016/j.arth.2008.10.013.

31. Peersman G, Laskin R, Davis J, et al. Infection in total knee replacement: a retrospective review of 6489 total knee replacements. Clin. Orthop. Relat. Res. 2001(392):15-23.

32. Jamsen E, Huotari K, Huhtala H, et al. Low rate of infected knee replacements in a nationwide series--is it an underestimate? Acta Orthop. 2009;**80**(2):205-12, doi: 10.3109/17453670902947432.

33. Deirmengian C, Kardos K, Kilmartin P, et al. Combined measurement of synovial fluid alpha-Defensin and C-reactive protein levels: highly accurate for diagnosing periprosthetic joint infection. J. Bone Joint Surg. 2014;**96**(17):1439-45, doi: http://dx.doi.org/10.2106/JBJS.M.01316.

34. Paxton EW, Inacio MC, Khatod M, et al. Risk calculators predict failures of knee and hip arthroplasties: findings from a large health maintenance organization. Clin. Orthop. Relat. Res. 2015;**473**(12):3965-73, doi: 10.1007/s11999-015-4506-4.

35. Kaier K, Mutters NT, Frank U. Bed occupancy rates and hospital-acquired infections--should beds be kept empty? Clin. Microbiol. Infect. 2012;**18**(10):941-5, doi: 10.1111/j.1469-0691.2012.03956.x.

For peer review only

36. Kurtz SM, Lau E, Schmier J, et al. Infection burden for hip and knee arthroplasty in the United States. J. Arthroplasty 2008;**23**(7):984-91, doi: 10.1016/j.arth.2007.10.017.

37. Bohl DD, Samuel AM, Basques BA, et al. How much do adverse event rates differ between primary and revision total joint arthroplasty? J. Arthroplasty 2015, doi: 10.1016/j.arth.2015.09.033.

38. Pugely AJ, Martin CT, Gao Y, et al. The incidence of and risk factors for 30-day surgical site infections following primary and revision total joint arthroplasty. J. Arthroplasty 2015;**30**(9 Suppl):47-50, doi: 10.1016/j.arth.2015.01.063.

39. Rasouli MR, Restrepo C, Maltenfort MG, et al. Risk factors for surgical site infection following total joint arthroplasty. J. Bone Joint Surg. Am. 2014;**96**(18):e158, doi: 10.2106/jbjs.m.01363.

40. Tayton ER, Frampton C, Hooper GJ, et al. The impact of patient and surgical factors on the rate of infection after primary total knee arthroplasty : an analysis of 64 566 joints from the New Zealand Joint Registry. Bone Joint J 2016;**98-B**(3):334-40, doi: 10.1302/0301-620X.98B3.36775.

41. Nichols CI, Vose JG. Clinical Outcomes and Costs Within 90 Days of Primary or Revision Total Joint Arthroplasty. J. Arthroplasty 2016, doi: 10.1016/j.arth.2016.01.022.

42. Mahomed NN, Barrett J, Katz JN, et al. Epidemiology of total knee replacement in the United States Medicare population. J. Bone Joint Surg. Am. 2005;**87**(6):1222-8.

43. Hartley RC, Barton-Hanson NG, Finley R, et al. Early patient outcomes after primary and revision total knee arthroplasty. A prospective study. J. Bone Joint Surg. Br. 2002;**84**(7):994-9.

44. Lavernia C, Lee DJ, Hernandez VH. The increasing financial burden of knee revision surgery in the United States. Clin. Orthop. Relat. Res. 2006;**446**:221-6.

45. Kallala RF, Vanhegan IS, Ibrahim MS, et al. Financial analysis of revision knee surgery based on NHS tariffs and hospital costs: does it pay to provide a revision service? Bone Joint J 2015;**97-B**(2):197-201, doi: 10.1302/0301-620x.97b2.33707.

46. Wolf CF, Gu NY, Doctor JN, et al. Comparison of one and two-stage revision of total hip arthroplasty complicated by infection: a Markov expected-utility decision analysis. J. Bone Joint Surg. Am. 2011;**93-A**(7):631-9.

47. Nagra NS, Hamilton TW, Ganatra S, et al. One-stage versus two-stage exchange arthroplasty for infected total knee arthroplasty: a systematic review. Knee Surg. Sports Traumatol. Arthrosc. 2015.

48. Jamsen E, Stogiannidis I, Malmivaara A, et al. Outcome of prosthesis exchange for infected knee arthroplasty: the effect of treatment approach. Acta Orthop. 2009;**80**(1):67-77, doi: 10.1080/17453670902805064.

For peer review only

49. Patel A, Pavlou G, Mujica-Mota RE, et al. The epidemiology of revision total knee and hip arthroplasty in England and Wales: a comparative analysis with projections for the United States. A study using the National Joint Registry dataset. Bone Joint J 2015;**97-B**(8):1076-81, doi: 10.1302/0301-620X.97B8.35170.

For peer review only

LIST OF ABBREVIATIONS

PJI: Prosthetic joint infection;

NJR: National Joint Registry for England, Wales and Northern Ireland

BMI: Body Mass Index

ASA grade: American Society of Anesthesiologists grade

RR: Rate Ratio;

95% CI: 95% Confidence Interval

ADDITIONAL FILES

Web appendix Table 1: Characteristics of patients with a completed or partially-completed two-stage revision total knee replacement

FIGURE LEGENDS

Figure 1: Description of procedures recorded in the National Joint Registry.

Figure 2: Prevalence (95% CI) of revision for prosthetic joint infection within 2 years from the index primary surgery.

Figure 3. Prevalence (95% CI) of revision for prosthetic joint infection within 2 years from the index aseptic revision surgery.

Figure 4 : Cumulative Incidence Function of revision for prosthetic joint infection following index primary and aseptic revision knee replacement.

ADDITIONAL FIGURE LEGENDS

Web Appendix Fig 1. Prevalence (95% CI) of revision for prosthetic joint infection within 2 to 6 years

For peer review only

from the index primary surgery.

Web appendix Fig 2. Prevalence (95% CI) of revision for prosthetic joint infection within 2 to 6 years from the index aseptic revision surgery.

For peer review only

For peer review only - http://bmjopen.bmj.com/site/about/guidelines.xhtml

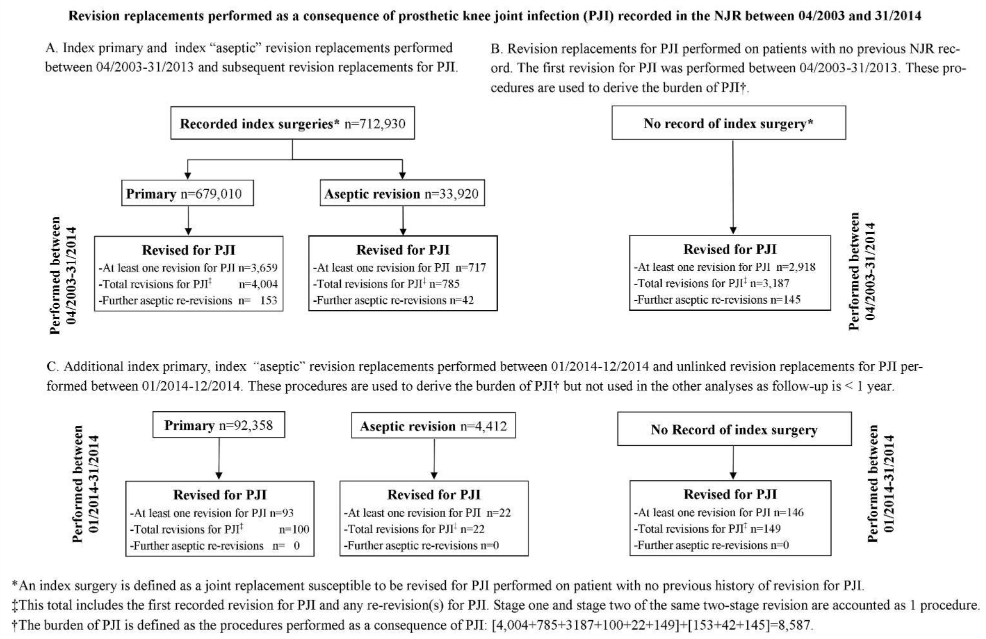

Figure 1
Figure 1
297x209mm (300 x 300 DPI)

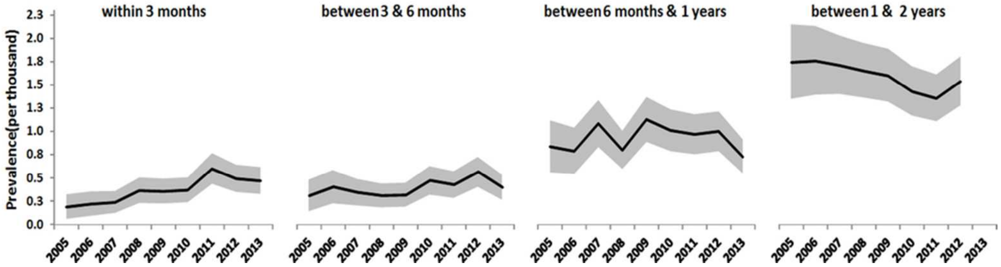

Figure 2
Figure 2
40x10mm (600 x 600 DPI)

For peer review only

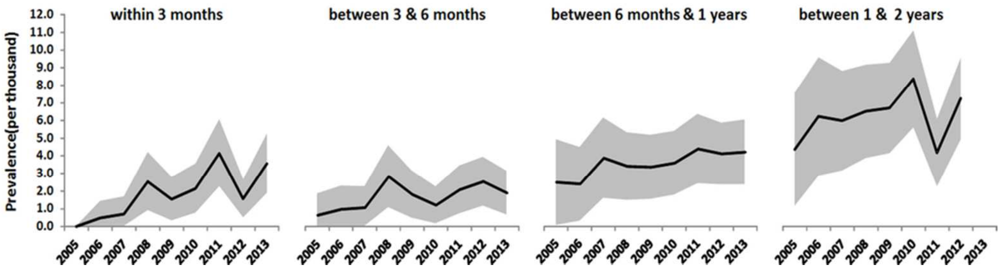

Figure 3

Figure 3

40x10mm (600 x 600 DPI)

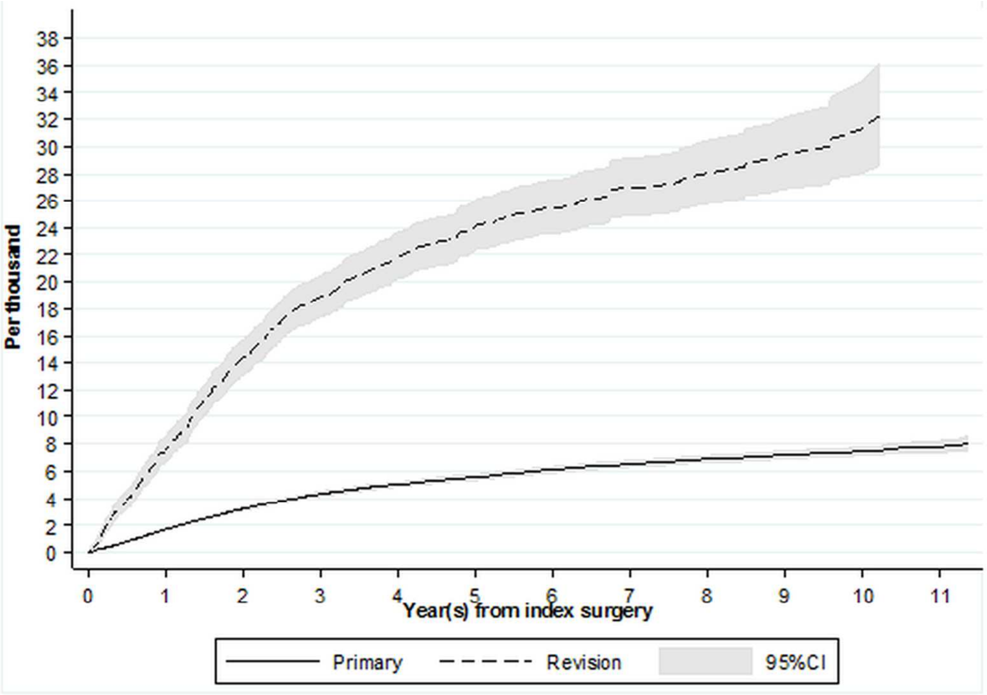

Figure 4
Figure 4
56x39mm (600 x 600 DPI)

For peer review only

Appendix Table 1: Characteristics of patients with a completed or partially-completed two-stage revision total knee replacement †

	Complete*	Only stage two**	Only stage one**
**Primary(n=2,880)**	1,512	792	576
Age(mean, SD)	66(9)	67(9)	67(10)
Female(%)	44	41	43
BMI(mean, SD)	32(6)	32(6)	32(6)
missing BMI (n=)	319	308	204
ASA 1(%)	14	14	10
ASA 2(%)	68	67	64
ASA 3(%)	18	19	25
ASA 4-5(%)	0	0	1
**Revision(n=589)**	309	154	126
Age(mean, SD)	66(10)	66(10)	68(10)
Female(%)	40	41	33
BMI(mean, SD)	32(6)	31(6)	32(6)
missing BMI	63	58	32
ASA 1(%)	14	10	14
ASA 2(%)	67	67	58
ASA 3(%)	19	22	27
ASA 4-5(%)	0	1	1

†2,880 two-stage revision total knee replacement procedures to manage an infection were recorded in the NJR following an index primary knee replacement and 589 following an index aseptic revision surgery.

* Both stage one and stage two procedures of a two-stage revision total knee replacement reported in the NJR.

**Only stage one or stage two procedure of a two-stage revision total knee replacement reported in the NJR.

For peer review only

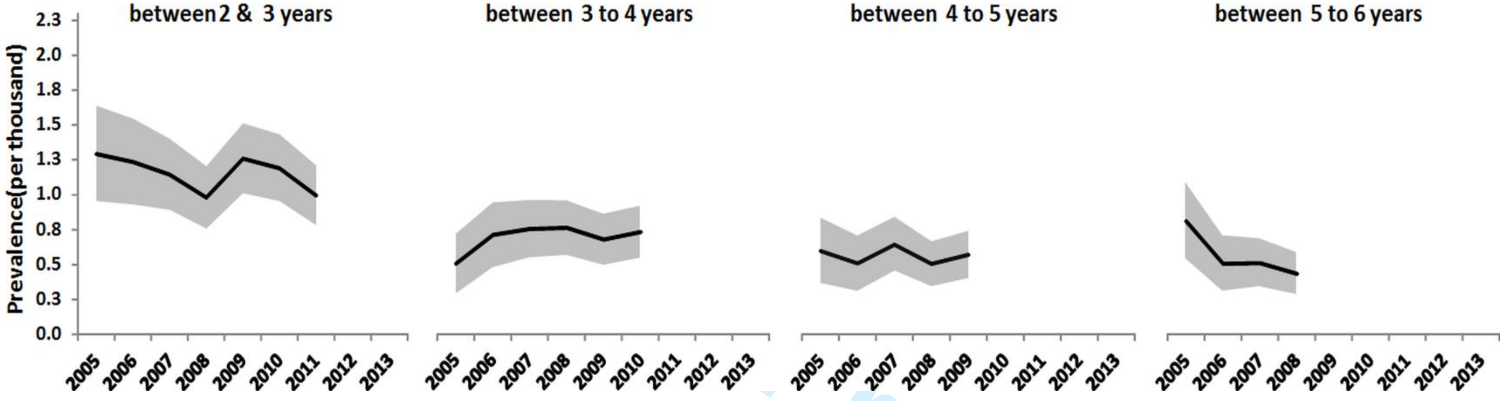

Web Appendix Fig 1. Prevalence (95% CI) of revision for prosthetic joint infection within 2 to 6 years from the index primary surgery.

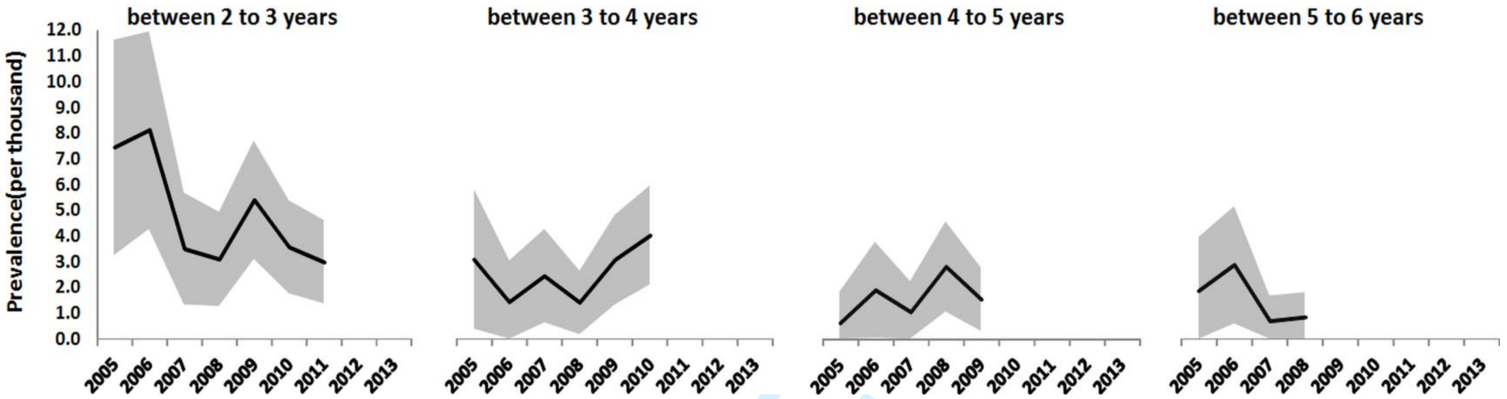

Web appendix Fig 2. Prevalence (95% CI) of revision for prosthetic joint infection within 2 to 6 years from the index aseptic revision surgery.

STROBE 2007 (v4) checklist of items to be included in reports of observational studies in epidemiology*

Checklist for cohort, case-control, and cross-sectional studies (combined)

Section/Topic	Item #	Recommendation	Checked
**Title and abstract**	1	(*a*) Indicate the study's design with a commonly used term in the title or the abstract	**Page 1**
		(*b*) Provide in the abstract an informative and balanced summary of what was done and what was found	**Pages 2-3**
**Introduction**			
Background/rationale	2	Explain the scientific background and rationale for the investigation being reported	**Pages 5-6**
Objectives	3	State specific objectives, including any pre-specified hypotheses	**Page 6**
**Methods**			
Study design	4	Present key elements of study design early in the paper	**Page 7**
Setting	5	Describe the setting, locations, and relevant dates, including periods of recruitment, exposure, follow-up, and data collection	**Pages 7-8+Figure 1**
Participants	6	Give the eligibility criteria, and the sources and methods of selection of participants. Describe methods of follow-up	**Page 7-8**
		For matched studies, give matching criteria and number of exposed and unexposed	na
Variables	7	Clearly define all outcomes, exposures, predictors, potential confounders, and effect modifiers. Give diagnostic criteria, if applicable	**Pages 7-8**
Data sources/ measurement	8*	For each variable of interest, give sources of data and details of methods of assessment (measurement). Describe comparability of assessment methods if there is more than one group	**Page 7-8**
Bias	9	Describe any efforts to address potential sources of bias	**Pages 8-9**
Study size	10	Explain how the study size was arrived at	**Page 7-8**
Quantitative variables	11	Explain how quantitative variables were handled in the analyses. If applicable, describe which groupings were chosen and why	**Pages 9-10**
Statistical methods	12	(*a*) Describe all statistical methods, including those used to control for confounding	**Pages 9-10**
		(*b*) Describe any methods used to examine subgroups and interactions	**Pages 9-10**
		(*c*) Explain how missing data were addressed	**Pages 8-9**
		(*d*) If applicable, explain how loss to follow-up was addressed	**Pages 8-9+Figure 1**
		(*e*) Describe any sensitivity analyses	na
**Results**			
Participants	13*	(a) Report numbers of individuals at each stage of study—eg numbers potentially eligible, examined for eligibility, confirmed eligible, included in the study, completing follow-up, and analysed	**Figure 1+Page 11**

For peer review only

		(b) Give reasons for non-participation at each stage	**Figure 1+Page 11**
		(c) Consider use of a flow diagram	**Figure 1+Page 11**
Descriptive data	14*	(a) Give characteristics of study participants (eg demographic, clinical, social) and information on exposures and potential confounders	**Page 11**
		(b) Indicate number of participants with missing data for each variable of interest	na
		(c)Summarise follow-up time (eg, average and total amount)	**Figures 2-4+Appendix figures 1-2+pages 10-13**
Outcome data	15*	Report numbers of outcome events or summary measures over time	**Tables 1-2+pages 11-14**
Main results	16	(*a*) Give unadjusted estimates and, if applicable, confounder-adjusted estimates and their precision (eg, 95% confidence interval). Make clear which confounders were adjusted for and why they were included	**Pages 11-14**
		(*b*) Report category boundaries when continuous variables were categorized	na
Other analyses	17	Report other analyses done—eg analyses of subgroups and interactions, and sensitivity analyses	**Pages 11-14**
**Discussion**			
Key results	18	Summarise key results with reference to study objectives	**Page 16**
Limitations	19	Discuss limitations of the study, taking into account sources of potential bias or imprecision. Discuss both direction and magnitude of any potential bias	**Pages 16-17**
Interpretation	20	Give a cautious overall interpretation of results considering objectives, limitations, multiplicity of analyses, results from similar studies, and other relevant evidence	**Pages 17-19**
Generalisability	21	Discuss the generalisability (external validity) of the study results	**Pages 17-18**
**Other information**			
Funding	22	Give the source of funding and the role of the funders for the present study and, if applicable, for the original study on which the present article is based	**Page 20**

*Give information separately for cases and controls in case-control studies and, if applicable, for exposed and unexposed groups in cohort and cross-sectional studies.

Note: An Explanation and Elaboration article discusses each checklist item and gives methodological background and published examples of transparent reporting. The STROBE checklist is best used in conjunction with this article (freely available on the Web sites of PLoS Medicine at http://www.plosmedicine.org/, Annals of Internal Medicine at http://www.annals.org/, and Epidemiology at http://www.epidem.com/). Information on the STROBE Initiative is available at www.strobe-statement.org.

For peer review only
